# CRISPR directed evolution of the spliceosome for resistance to splicing inhibitors

**DOI:** 10.1186/s13059-019-1680-9

**Published:** 2019-04-30

**Authors:** Haroon Butt, Ayman Eid, Afaque A. Momin, Jeremie Bazin, Martin Crespi, Stefan T. Arold, Magdy M. Mahfouz

**Affiliations:** 1 Laboratory for Genome Engineering and Synthetic Biology, King Abdullah University of Science and Technology (KAUST), Thuwal, Saudi Arabia; 2 Division of Biological and Environmental Sciences and Engineering, Computational Bioscience Research Center, KAUST, Thuwal, Saudi Arabia; 3 CNRS, INRA, Institute of Plant Sciences Paris-Saclay IPS2, Univ Paris Sud, Univ Evry, Univ Paris-Diderot, Sorbonne Paris-Cite, Universite Paris-Saclay, Orsay, France

**Keywords:** Directed evolution, Genome engineering, Spliceosome, SF3B complex, SF3B1, Splicing modulators, Herboxidiene, Pladienolide B, Spliceostatin A, CRISPR/Cas9, Herbicide resistance

## Abstract

Increasing genetic diversity via directed evolution holds great promise to accelerate trait development and crop improvement. We developed a CRISPR/Cas-based directed evolution platform in plants to evolve the rice (*Oryza sativa*) SF3B1 spliceosomal protein for resistance to splicing inhibitors. SF3B1 mutant variants, termed SF3B1-GEX1A-Resistant (SGR), confer variable levels of resistance to splicing inhibitors. Studies of the structural basis of the splicing inhibitor binding to SGRs corroborate the resistance phenotype. This directed evolution platform can be used to interrogate and evolve the molecular functions of key biomolecules and to engineer crop traits for improved performance and adaptation under climate change conditions.

**Electronic supplementary material:**

The online version of this article (10.1186/s13059-019-1680-9) contains supplementary material, which is available to authorized users.

## Background

Technologies for targeted and accelerated improvement of crop traits are urgently needed to increase crop yield and meet the demands of the burgeoning world population [1–4]. In directed evolution, genetic diversity is artificially increased to produce protein variants; this is followed by screening and selection for functional variants with improved fitness [5]. Directed evolution via targeted sequence diversification and selection has revolutionized our ability to develop diverse biomolecules with novel and improved functions for various applications in basic biology and biotechnology.

Most directed evolution approaches have been developed in bacteria or yeast. However, the functions of evolved biomolecules are best tested in their native cellular context to avoid serious drawbacks including stability and activity issues [6]. Therefore, the use of complex eukaryotes as hosts for the directed evolution of biomolecules improves the chances of successful selection of the sought-after variant for the intended application. Directed evolution platforms in mammalian cells include plasmid mutagenesis, followed by transfection and screening, in vivo mutagenesis via somatic hypermutations in immune cells, robotic cell-picking technologies, and error-prone DNA replication in the gene of interest [7–9]. The clustered regularly interspaced palindromic repeats (CRISPR)/Cas9 system has been harnessed for genome editing applications across diverse eukaryotic species including plants. CRISPR/Cas9 generates a site-specific double-strand break (DSB) at a user-defined DNA sequence. This DSB can be repaired via the precise homology-directed repair (HDR) or error-prone nonhomologous end joining (NHEJ) repair mechanism thus producing a variety of genetic outcomes, including single-nucleotide changes and large or small genetic deletions [10–12]. Very recently, cytidine deaminases and DNA polymerases fused to Cas9 variants have been used for localized diversification of gene sequences. This enables directed evolution for improved functions capitalizing on the bimodular function of these chimeric proteins, where Cas9 serves as a targeting module and the cytidine deaminase of DNA polymerase produces localized sequence changes in the targeted user-defined region [13–15]. Despite these advances in mammalian systems, directed evolution in plants remains an underexplored field.

Developing directed evolution platforms in plants may help identify novel traits, expand the range of traits, and accelerate trait development and improvement, which are crucial for maximizing the genetic potential of crop plants and their resilience to climate change [16]. In this work, we developed and employed a CRISPR/Cas-based directed evolution platform to evolve the rice SF3B1 spliceosomal protein for resistance to splicing inhibitors.

## Results and discussion

### CRISPR/Cas-directed evolution platform

Applications of CRISPR/Cas9 in plants are mainly focused on the generation of functional knockouts via targeted DSB formation and efficient NHEJ, because HDR is quite inefficient in plants. To develop an efficient directed evolution platform in plants, we used a combination of targeted mutagenesis and selection. To this end, we designed a CRISPR/Cas-directed evolution (CDE) platform employing CRISPR/Cas9 to generate DSBs at all possible coding sequence sites in a specific gene of interest. CRISPR/Cas9 requires a protospacer adjacent motif (PAM) of NGG for cleavage; therefore, only sequences adjacent to a PAM can be targeted.

**Fig. 1:**
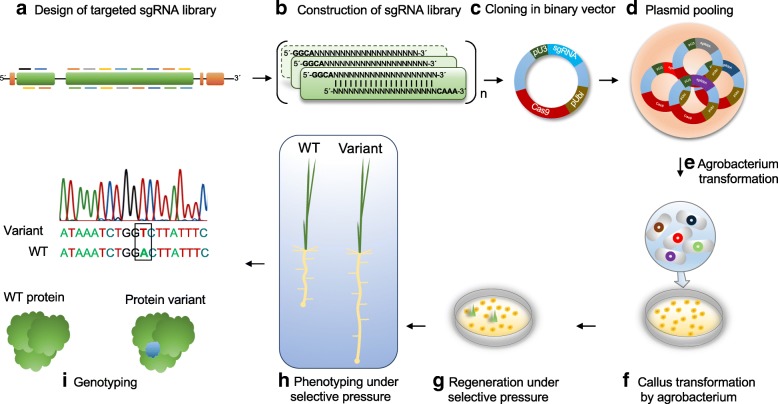
The CRISPR/Cas-directed evolution (CDE) platform. **a** All possible sgRNAs targeting the whole coding sequence of a gene are designed. **b** The sgRNA library is constructed via oligo synthesis and annealing. **c** The annealed oligos are cloned with sgRNA scaffold under the *U3* promoter in the binary vector. The sequences are confirmed by Sanger sequencing. **d** All the plasmids are pooled in equimolar ratios. **e** The pooled plasmids are transformed into *Agrobacterium*. **f** The *Agrobacterium* cells are washed from plates with transformation medium and used for callus transformation. **g** After two consecutive selections on hygromycin, the callus is regenerated under selection pressure (e.g., splicing inhibitor). **h** The resistant seedlings are recovered. **i** The resistant plants are further analyzed by exhaustive phenotyping under selection pressure. The plants are genotyped by amplicon sequencing, and protein variants are identified

For CDE, a targeted library of single guide RNAs (sgRNAs) is designed, cloned into a binary vector, and then transformed into *Agrobacterium tumefaciens* for stable plant transformation. Agrobacteria harboring the sgRNA library are used for plant transformation, and plants are regenerated under selective pressure to force accelerated evolution. This enables the recovery of protein variants that give the plant the ability to survive under selective pressure. Plant regenerants that survive the selective pressure are genotyped and their progeny phenotyped to link genotype to phenotype and examine mutant variants of the protein (Fig. 1). The ability to segregate out the CRISPR-Cas9 machinery and recover progeny plants harboring the evolved variant of interest without any foreign DNA makes this an elegant system for directed evolution applications.

### CRISPR/Cas directed evolution of SF3B1

To provide proof of concept for CDE, we evolved the spliceosome component SF3B1 for resistance to splicing inhibitors. In plants, naturally occurring splicing inhibitors, including pladienolide B (PB) and herboxidiene (GEX1A), have massive effects on the splicing machinery, resulting in transcriptome-wide splicing repression [17, 18]. These polyketide natural products, produced by *Streptomyces* sp*.*, have been applied to plants as herbicides and to mammalian cells as anti-cancer therapies [19–21]. Work in mammalian cells showed that these splicing inhibitors target the core splicing factor SF3B1, of the SF3B complex of the U2 snRNP of the spliceosome [22]. We have tested the effects of PB and GEX1A on *Oryza sativa* (rice) seed germination and primary root (PR) length, and our data showed that these splicing modulators significantly inhibit seed germination and PR growth in a dose-dependent manner (Additional file 1: Figure S1).

Due to the strong conservation with the mammalian SF3B complex, we hypothesized that the rice SF3B complex is targeted by these splicing inhibitors. We searched the rice genome for SF3B genes and found that OsSF3B1 is highly conserved compared with its mammalian homolog (Additional file 1: Figure S2). To test our CDE platform on the evolution of SF3B1 resistance to splicing inhibitors, we used the CRISPR/Cas9 system to generate SF3B1-resistant variants. Because splicing inhibition leads to inhibition of plant growth and development, we selected plants during regeneration of callus using a splicing inhibitor to identify resistant variants.

**Fig. 2:**
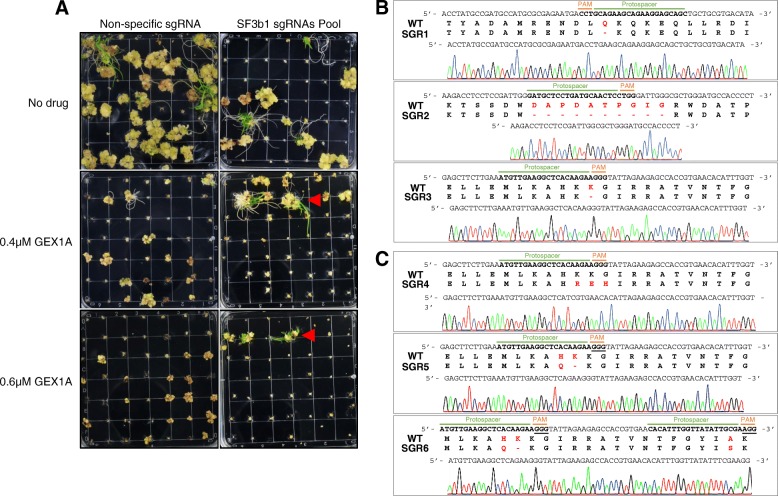
Generation of SF3B1 variants using the CDE platform. **a** *Agrobacterium*-mediated transformation was conducted using the sgRNA library targeting *SF3B1*. After selection on hygromycin, regeneration was performed under selection pressure of GEX1A (0.4 or 0.6 μM). A non-specific sgRNA was transformed and used as GEX1A selection control. Regeneration was only observed with the sgRNA library targeting *SF3B1*. Red arrows indicate the GEX1A-resistant shoots. **b** The resistant plants genotyped by Sanger sequencing and revealed in-frame mutations in *SF3B1*. These mutants were named SGR (SF3B1 GEX1 Resistant). The red letters indicate the amino acids modified in mutant sequence. SGR1 has a deletion of Q157. SGR2 has a deletion of ten amino acids DAPDATPGIG (223–232). SGR3 has a deletion of K1050. The chromatograms show Sanger sequencing of *SF3B1* mutant variants. **c** A protein domain-focused CDE platform used to generate SF3B1 mutant variants resistant to GEX1A. SGR4 has three consecutive substitutions K1049R, K1050E, and G1051H. SGR5 has the H1048Q substitution and K1049 deletion. SGR6 has the H1048Q substitution, K1049 deletion, and A1064S substitution. SGR4 and SGR5 were recovered with the sgRNA HR target while SGR6 was recovered with PTG transformation

To generate SF3B1 variants, we designed 119 sgRNAs targeting all possible PAM-adjacent sites in the whole coding sequence (CDS) of *SF3B1* (Additional file 2: Table S1). The sgRNA library was constructed via oligonucleotide synthesis and annealing of all possible targets in *SF3B1* into the sgRNA scaffold, under the control of *U3* promoter in the binary vector pRGEB32 (Additional file 2: Table S2). Cas9 was produced under the control of the *OsUbiquitin* promoter on the same plasmid. The plasmids of this library were pooled and transformed into the *Agrobacterium tumefaciens* EHA105 strain for stable transformation of rice (cv. Nipponbare) embryonic callus. Subsequently, we performed callus transformation and selection for stable transformation. From the transformed callus, we regenerated whole plants on different concentrations of GEX1A. We used different concentrations to provide variable levels of selective pressure to trigger NHEJ repair and generation of SF3B1 variants resistant to GEX1A. We sub-cultured 15,000 transformed calli onto medium supplemented with 0.4 μM and 0.6 μM GEX1A, concentrations that are sufficient to inhibit wild-type callus growth. We recovered 21 rice shoots on 0.4 μM GEX1A (Additional file 2: Table S4). Our data thus showed that our directed evolution platform was successful in regenerating GEX1-resistant seedlings that were likely to possess an *SF3B1* mutation (Fig. 2a).

To confirm that these seedlings carried mutations in the *SF3B1* gene, we identified the sgRNA sequence in each resistant seedling through PCR amplicon sequencing. This allowed us to determine the target sequence in the *SF3B1* gene for genotyping and to test the presence, nature, and identity of the causal mutation (Fig. 2b). We selected several mutants, including *SGR1* (SF3B1-GEX1A-Resistant), *SGR2*, and *SGR3* for further study and analysis (Fig. 2b). Our sgRNA sequencing data revealed that these seedlings carried a single sgRNA. To determine the mutations triggered by these sgRNAs, we PCR-amplified the region encompassing the target sequence from T_0_ plants and subjected these PCR amplicons to Sanger sequencing.

Our data showed that the GEX1A-resistant seedlings possessed in-frame *SF3B1* mutations that are likely to be functional to support RNA splicing, but could affect drug binding (Fig. 2b). For example, the *SGR1* mutant had a 3-bp mutation causing a predicted single amino acid deletion of Q157. The *SGR2* mutant had a deletion of 10 amino acids, DAPDATPGIG (amino acids 223 to 232). *SGR3* had a single amino acid deletion of K1050; mutation in the same K1071 of the human homolog HsSF3B1 confers resistance to splicing inhibitors [21]. In an experiment without GEX1A selection, we tried to recover knockout mutants of *SF3B1*. In the T_0_, we recovered seedlings harboring a heterozygous single-nucleotide deletion mutation with sgRNA-18 and an in-frame mutant with sgRNA-50 causing a 15 amino acid deletion LPLMKPEDYQYFGTL (442–456). The heterozygous knockout mutant was not heritable in the seed progeny. However, the in-frame mutant variant with the 15 amino acid deletion exhibited no resistance to GEX1A (Fig. 2 and Additional file 1: Figure S4). Because we were unable to recover any functional knockout (out-of-frame) mutants of *SF3B1*, we concluded that the loss of functional SF3B1 is embryonic lethal and that this is an essential gene for seed viability.

### Domain-focused directed evolution

We then tested whether domain-focused directed evolution, in which targeted mutagenesis is conducted on key protein domains known to mediate key interactions for function, stability, or activity, was achievable in our CDE platform. We examined whether targeting SF3B1 protein domains, known to mediate drug–protein interactions, in our CDE platform could generate SF3B1 variants resistant to GEX1A. We capitalized on our prior knowledge of the key SF3B1 domains that mediate drug interactions and spliceosome inhibition and designed sgRNAs targeting these domains (Additional file 1: Figure S3). HEAT repeats (HR) 15–17 are highly conserved (Additional file 1: Figure S2 and S3) and splicing inhibitor-resistant mutations identified in mammalian cell cultures clustered around this region [21]. We targeted this region by using a single sgRNA (HR-target) or a PTG (polycistronic tRNA-gRNA) fragment that contained two sgRNAs (Additional file 1: Figure S3). After application of our CDE platform and rice callus transformation, we recovered SF3B1 variants highly resistant to GEX1A, including *SGR3* to *SGR6* (Fig. 2c, Additional file 1: Figure S5 and Additional file 2: Table S3). The *SGR3* mutant was also recovered from GEX1A-resistant seedlings of a single sgRNA (Fig. 2a). Our data show that, compared with sgRNAs targeting random regions, the sgRNAs targeting HR15–17 are more effective in producing rice seedlings capable of survival on GEX1A-supplemented media. Genotyping analysis revealed that these mutations included *SGR4* with three amino acid substitutions (K1049R, K1050E, and G1051H). *SGR5* had a substitution (H1048Q) and a deletion (K1049). SGR6 was recovered from PTG transformation and had two substitutions (H1048Q and A1064S), and a deletion (K1049, Fig. 2c).

To test the heritability of these mutations and the survival of the homozygous SF3B1 mutant variants, we conducted a phenotypic and genotypic analysis of the seed progeny of the *SGR1, SGR2, SGR3*, *SGR4*, *SGR5*, and *SGR6* mutants. Genotyping analysis revealed that these mutants were heritable in a homozygous fashion, indicating that these SF3B1 variants support efficient splicing, because these mutants are phenotypically indistinguishable from wild-type plants (Additional file 1: Figure S6).

### SF3B1 mutant variants confer variable resistance to GEX1A

**Fig. 3:**
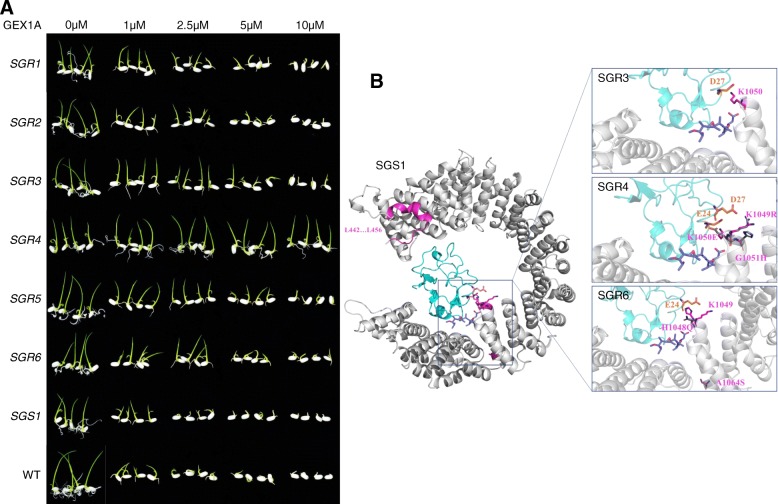
SGRs confer resistance to GEX1A treatment and the structural basis of resistance. **a** Dose–response effects of GEX1A treatment on germination of WT, SGS, SGR1, SGR2, SGR3, SGR4, SGR5, and SGR6 seeds. Rice seeds were sterilized and germinated in dH_2_O with different concentrations of GEX1A. Hypocotyl emergence was considered as germination. We observed that the rice seed germination was affected in a dose-dependent manner (Additional file 1: Figure S1). Germination of wild-type and SGS1 seeds was severely inhibited at 2.5 and 5 μM GEX1A. Germination of SGRs is less affected by GEX1A treatment. SGR4 germination is completely unaffected even at 10 μM GEX1A (*n* = 8). **b** The structural basis of the SF3B1 mutant variant resistance to GEX1A. The 3D model for the OsSF3B1:PHF5A complex and GEX1A with OsSF3B1 is represented in gray, PHF5A is represented in cyan, and GEX1A represented in blue. Key residues on PHF5A are shown in orange whereas residues for wild type are shown in magenta and mutations on OsSF3B1 are shown in gray

Next, we analyzed the responses of SGRs to GEX1A treatments. Therefore, we conducted germination and root inhibition assays at several GEX1A concentrations (Fig. 3 and Additional file 1: Figures S7 and S8). As indicated earlier, GEX1A affected rice germination at 2.5 μM and severely inhibited seed germination at 5 μM (Additional file 1: Figure S1). Here, we observed that the germination of all SGRs was not affected at 2.5 μM GEX1A but the germination of *SGS1* and the wild-type rice seeds was inhibited (Fig. 3 and Additional file 1: Figures S7 and S8). When we increased the GEX1A concentration to 10 μM, the germination of all SGRs was inhibited except SGR4, which was completely unaffected by GEX1A (Fig. 3 and Additional file 1: Figures S7 and S8). Similar results were observed for the root inhibition assay (Additional file 1: Figure S8), where all the SGRs showed resistance to 0.3 and 0.5 μM GEX1A. However, the root growth of *SGS1* and wild-type seedlings completely ceased at 0.3 μM of GEX1A (Additional file 1: Figure S8). Intriguingly, root growth was inhibited for all SGRs at 1 μM GEX1A, except SGR4, which did not exhibit root growth inhibition at any concentration. These data indicate that the SF3B1 variants exhibit variable degrees of resistance to GEX1A, with SGR4 conferring the strongest resistance.

Several reports have indicated that GEX1A, PB, and Spliceostatin A share the same pharmacophore and bind to the same site in the SF3B1 protein [23, 24]. To investigate whether GEX1A and PB share the same binding site in SF3B1, we tested whether GEX1A-resistant mutants were also resistant to PB by conducting primary root growth assays on media supplemented with PB (Additional file 1: Figure S9). SF3B1 mutant variants were resistant to PB (Additional file 1: Figure S9). The in-frame sensitive GEX1A mutant was also sensitive to PB. These data provide compelling evidence that these splicing drugs target the same SF3B1 domain.

### Structural basis of SGR resistance to GEX1A

Recent reports have provided extensive structural studies on mammalian SF3B1 and the SF3B1:PHF5 complex and their interaction with various splicing inhibitors [24, 25]. Since rice and mammalian SF3B1 are > 80% identical, we used these mammalian structures to study the structural basis of the resistance of the rice SF3B1 mutant variants to GEX1A and PB (Fig. 3b, Additional file 1: Figures S10 and S11). Based on these structural models, we assessed the molecular effect of six OsSF3B1 variants on interactions with PB and herboxidiene. The SGS1 variant results in the deletion of an N-terminal loop-helix element (Fig. 3b, Additional file 1: Figure S11). Although this deletion might mildly reduce protein stability, it is remote from the drug binding site and hence should not lead to drug resistance, in agreement with our experimental observations. The SGR3 K1050 variant is located at the rim of the drug binding surface and reinforces PHF5A interactions through an ionic bond to PHF5A D27 (Fig. 3b, Additional file 1: Figure S11). Deletion of K1050 perturbs the binding pocket’s stereochemistry and weakens contacts to PHF5A.

The SGR4 variant contains two mutations that are not predicted to have marked effects: K1049R is a substitution with similar physico-chemical properties, and although G1051H leads to inclusion of a much larger side chain, this change does not cause steric clashes, being exposed to the solvent (Fig. 3b, Additional file 1: Figure S11). Conversely, K1050E will lead to electrostatic repulsion with GEX1A, weakening the drug binding interactions, and additionally introduce a repulsion with PHF5A D27 and weaken the OsSF3B1:PHF5A interaction.

In the SGR6 variant (H1048Q, K1049 deletion, A1064S), H1048 and K1049 are located at the rim of the drug binding site, at the interface between OsSF3B1 and PHF5A (Fig. 3b, Additional file 1: Figure S11). The double mutation H1048Q/K1049del will significantly destabilize the drug binding pocket and affect the binding to PHF5A. The third mutation A1064S is located 14 Å away from the drug binding site, and the relatively homologous substitution with a serine would only lead to minor local structural rearrangements.

The SGR Q157 deletion and DAPDATPGIG (223–232) variants carry mutations in the disordered loop region in the N-terminal of the protein, not included in our molecular models (Additional file 1: Figure S10). We hypothesize that these deletions might affect additional interactions or regulatory roles that this region might have. Collectively, our structural analysis demonstrated that the drug-resistant variants contained driver and passenger mutations and that much of the effect was mediated through an OsSF3B1 region that is at the intersection between drug and PHF5A binding. Thus, the OsSF3B1 mutations appeared to have a double effect by destabilizing binding of both drug and PHF5A. We anticipate that these data will be useful to predict and identify drug resistance in various applications.

### SGR4 mutant variant exhibits efficient splicing in GEX1A treatment

**Fig. 4:**
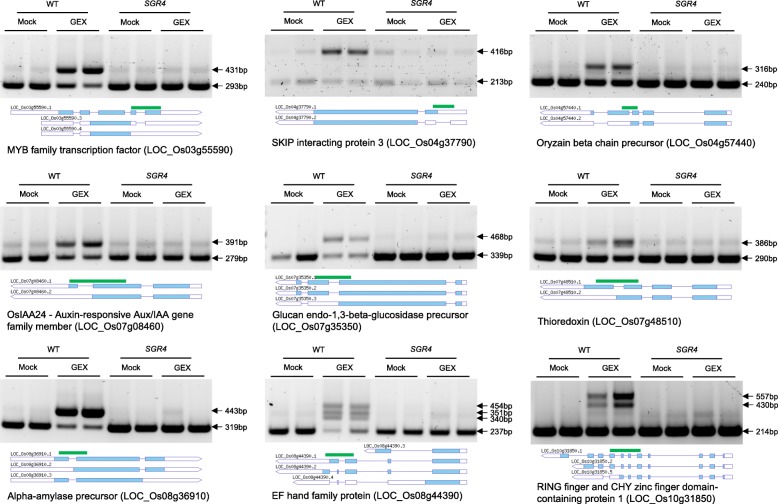
GEX1A treatment inhibits pre-mRNA splicing in WT rice but not the SGR4 mutant. The cDNAs were prepared from 1-week-old rice seedlings that were treated with 0.3 μM GEX1A for 6 h. RT-PCR was performed using primers that flank alternatively spliced introns in selected genes. Arrowheads indicate splicing variants that changed following GEX1A treatment. The gene structure flanking the amplified fragments and the structures of regulated variants are shown under the gel images (the green line marks the positions of the PCR product). Blue box, exon; line, intron; white box, 5′ or 3′ UTR. The gene locus identifier is shown on the bottom

GEX1A and PB inhibit splicing and cause significant intron retention in *Arabidopsis thaliana*. Therefore, we wanted to test the effects of GEX1A on the seedlings of wild-type rice and SF3B1 mutant variants and examine their molecular effects on splicing repression. Subsequently, we treated 1-week-old wild-type rice seedlings and SF3B1 variants with 0.3 μM GEX1A for 6 h. After extracting the RNA, cDNA was synthesized and semi-quantitative PCR was conducted on exons flanking selected introns. We have tested several genes with alternatively spliced pre-mRNAs, including those encoding: MYB family transcription factor (LOC_Os03g55590), SKIP interacting protein 3 (LOC_Os04g37790), Oryzain beta chain precursor (LOC_Os04g57440), Glucan endo- 1,3-beta-glucosidase precursor (LOC_Os07g35350), EF hand family protein (LOC_Os08g44390), and Alpha-amylase precursor (LOC_Os08g36910), and RING finger and CHY zinc finger domain-containing protein 1 (LOC_Os10g31850) to test whether they exhibit intron retention upon GEX1A treatments. Our data show that the GEX1A treatment enhanced the intron retention events during pre-mRNA splicing of these genes in wild-type rice seedlings (Fig. 4).

Because the SGR4 variant exhibited very strong resistance to GEX1A treatment in the germination and root-length assays, we treated 1-week-old seedlings of *SGR4* with 0.3 μM GEX1A for 6 h and conducted RT-PCR to determine intron retention patterns. Interestingly, our data show no intron retention events for plants carrying the SGR4 variant (Fig. 4) further corroborating our phenotyping and structural analysis data. These data validate our hypothesis and the CDE platform and indicate that GEX1A is not capable of binding to the SF3B1 SGR4 variant, resulting in proficient pre-mRNA splicing.

## Conclusion

Our study has demonstrated the power of the CDE platform to enable engineering of the spliceosome, which may serve as a target to inspire efforts to develop a novel class of herbicides with biotechnological applications in agriculture [19]. Furthermore, this work indicates that plants provide an attractive and inexpensive system for chemical genomics and directed evolution approaches and may aid in the discovery of clinically valuable splicing modulators [20].

Although we used targeted mutagenesis and harnessed NHEJ to generate variants with improved functions, we envision that other CRISPR/Cas-based sequence diversification platforms, including CRISPR-X and EvlovR, coupled with selective pressure may be useful in future CDE platforms [26–28]. Our CDE platform in plants explores the mutation space rapidly, within the short time it takes for plant regeneration. Our CDE platform can be applied to key genes controlling plant responses to abiotic or biotic factors to produce variants with improved abilities to survive harsh and adverse environments [29, 30]. Because plant cells are amenable to various manipulations and treatments at different growth and developmental stages, employing the CDE platform with exposure to various biotic and abiotic factors as selective pressure holds great promise for crop trait engineering to develop resistance to various biotic and abiotic factors to enhance food security. It is worth noting that this platform can be applied to single genes, multi-gene pathways, or gene networks. In this work, we delivered the sgRNA library via *Agrobacterium*, but a targeted synthetic sgRNA library produced as RNA–Cas9 ribonucleoprotein complexes can be delivered to simultaneously expand the sequences targeted for mutation. Moreover, while this platform focuses on protein evolution, other types of biomolecules could be targeted for CDE applications in plants and other eukaryotes.

This work provides an exciting proof of concept on the use of the CRISPR/Cas9 system in plants for directed evolution, offering many possibilities for trait engineering and breeding of crops with enhanced or optimum performance under climate change.

## Methods

### Plant materials and vector construction

*Oryza sativa* L. ssp. *japonica* cv. Nipponbare was used for all experiments. The expression of Cas9 was driven by *OsUbiquitin*, and the sgRNA was expressed under the *OsU3* promoter*.* The sgRNAs were designed to cover most of the genomic region of *SF3B1* (LOC_Os02g05310; Additional file 2: Table S1). The *pRGEB32* plasmid was digested with *Bsa*I, sgRNAs were synthesized as oligonucleotides with *Bsa*I overhangs, GGCA in the forward oligonucleotides and AAAC in the reverse (Additional file 2: Table S2). The oligonucleotides were annealed and ligated in the *Bsa*I-digested vector. For domain-focused evolution, sgRNA-92, sgRNA-119, and a PTG fragment with two sgRNAs (92 and 119) were cloned.

### Growth inhibition activities of GEX1A and PB

The germination inhibition assays were performed in six-well culture plates. Rice seeds were surface-sterilized and germinated in 4 mL of H_2_O for controls and with serial dilutions of GEX1A and PB for the treatments. The germination rate was calculated after 5 days. For root inhibition assays, sterilized rice seeds were germinated on half-strength Murashige and Skoog (½ MS) media (without sucrose) on square plates in a vertical position. After germination, 3-day-old seedlings were transferred to ½ MS plates supplemented with different concentrations of GEX1A and PB. The position of the root tip was marked, and the root inhibition rate was calculated after 3 days.

### Rice transformation

*Agrobacterium*-mediated rice transformation was performed as described previously [15]. All the plasmids were pooled in equimolar ratios and transformed into *Agrobacterium tumefaciens* EHA105. The colonies were washed from the plates with transformation solution (AAM media) and the OD_600_ adjusted to 0.3. Around 20,000 calli were transformed with *Agrobacterium*. After two rounds of selection on hygromycin, calli were regenerated with or without 0.4 μM and 0.6 μM GEX1A. A plasmid containing a non-specific sgRNA against *SF3B1* was used in a control transformation.

### SF3B1 mutants screening for GEX1A resistance

Seeds were harvested from T_0_ GEX1A-resistant plants. The seeds were de-husked, sterilized, and grown vertically on square plates containing ½ MS media (without sucrose). Wild-type and non-specific sgRNA seeds were used as controls. After 3 days, seedlings with similar root growth were transferred to ½ MS plates supplemented with 0.3 μM GEX1A. The root tips were marked to observe growth. The seedlings were grown vertically for another 3 days and then imaged. The resistant seedlings (T_1_) were transferred to soil.

### Genotyping of the SF3B1 mutant plants

Transgenic rice plants were grown in a greenhouse in soil at 28 °C. After 1 week, when plants were established on soil, DNA was extracted. For GEX1A-resistant T_0_ plants produced from the CDE platform, first T-DNA-specific PCR was performed and the products sequenced by Sanger sequencing. Then, the sgRNA targeted genomic region was amplified by PCR using specific primers for locus LOC_Os02g05310. Purified PCR products were cloned into CloneJET PCR Cloning Kit (K1231). Sanger sequencing was performed to analyze the mutations.

### Construction of a 3D model for the OsSF3B1–OsPHF5A complex

Homology models of the structures of the complex formed by OsSF3B1 (residues 441–1280) and OsPHF5A were produced based on the crystal structure of the complex of the human orthologues (PDB id: 6en4; 82% and 91% sequence identity, respectively), using SwissModel. RaptorX and IUPred2 were used to predict protein disorder. The compounds herboxidiene and pladienolide B (PB) were downloaded from the ZINC database in SD format and were converted to PDB format using OpenBabel 2.3.1. Flexible docking of the ligand compounds to the protein complex was performed using AutoDock 4.2. The grid parameters for the docking search were limited to cover the binding pocket identified in the human orthologue (PDB ID 6en4). Docking was performed using AutoDock default parameters. All the models and protein mutations were manually evaluated using the PyMOL program (pymol.org).

### RNA isolation and semi-quantitative RT-PCR

Total RNA was extracted from rice seedlings after 6 h of treatment with DMSO or 0.3 μM GEX1A using the Direct-zol RNA MiniPrep Plus (Zymo Research) according to the manufacturer’s recommendations. For reverse-transcription PCR (RT-PCR), DNA digestion of total RNA samples was performed using an RNase-Free DNase Set (Invitrogen cat. No. 18068-015) following the manufacturer’s protocol. The total RNA was reverse transcribed using a SuperScript First-Strand Synthesis System (Invitrogen) to generate cDNA. PCR conditions were initial denaturation at 95 °C for 2 min then 40 cycles of 95 °C for 30 s, 55 °C for 30 s, 72 °C for 60 s then final elongation is 72 °C for 5 min. Primers used for RT-PCR are listed in Additional file 2: Table S5.

## Additional files

Additional file 1: **Figure S1.** GEX1A and PB splicing modulators inhibit rice seed germination and primary root growth. **Figure S2.** Conservation of SF3B1 in eukaryotes. **Figure S3.** Domain-focused directed evolution of SF3B1. **Figure S4.** Genotyping of *SGS1.* **Figure S5.** Generation of SF3B1 variants using domain-focused CDE platform. **Figure S6.** Analysis of Seed progeny of SF3B1 mutant variants. **Figure S7.** SGRs confer resistance to GEX1A treatment. **Figure S8.** SGRs primary root growth is not inhibited by GEX1A treatment. **Figure S9.** SGRs primary root growth is not inhibited by PB treatment. **Figure S10.** The SF3B1 protein disorder plot and surface representation of 3D structure. **Figure S11.** The structural basis of SGRs resistance to PB. (PDF 2492 kb)

Additional file 2: **Table S1.** Sequences of sgRNAs with PAM targeting SF3B1. **Table S2.** List of primers annealed to synthesis sgRNAs with *Bsa*I compatible overhangs. **Table S3.** List of primers used for genotyping. **Table S4.** CDE platform for SF3B1 resistance to splicing inhibitor **Table S5.** List of primers used for intron retention analysis. (PDF 288 kb)
