# Supplementary material for: CRISPR directed evolution of the spliceosome for resistance to splicing inhibitors
Source: Genome Biol. 2019 Apr 30;20:73. doi: 10.1186/s13059-019-1680-9 (PMC6489355; doi:10.1186/s13059-019-1680-9)
Supplement: Supplementary file 1 — Figure S1. GEX1A and PB splicing modulators inhibit rice seed germination and primary root growth. Figure S2. Conservation of SF3B1 in eukaryotes. Figure S3. Domain-focused directed evolution of SF3B1. Figure S4. Genotyping of SGS1. Figure S5. Generation of SF3B1 variants using domain-focused CDE platform. Figure S6. Analysis of Seed progeny of SF3B1 mutant variants. Figure S7. SGRs confer resistance to GEX1A treatment. Figure S8. SGRs primary root growth is not inhibited by GEX1A treatment. Figure S9. SGRs primary root growth is not inhibited by PB treatment. Figure S10. The SF3B1 protein disorder plot and surface representation of 3D structure. Figure S11. The structural basis of SGRs resistance to PB. (PDF 2492 kb) [file 13059_2019_1680_MOESM1_ESM.pdf]

Additional file 1

CRISPR-directed evolution of the spliceosome for resistance to splicing inhibitors

Haroon Butt[1], Ayman Eid[1], Afaque A. Momin[2], Jeremie Bazin[3], Martin Crespi[3], Stefan T. Arold[2], and Magdy M. Mahfouz[1,*]

[1]Laboratory for Genome Engineering and Synthetic Biology, King Abdullah University of Science and Technology (KAUST), Thuwal, Saudi Arabia, [2]Division of Biological and Environmental Sciences and Engineering, Computational Bioscience Research Center, KAUST, Thuwal, Saudi Arabia, and [3]CNRS, INRA, Institute of Plant Sciences Paris-Saclay IPS2, Univ Paris Sud, Univ Evry, Univ Paris-Diderot, Sorbonne Paris-Cite, Universite Paris-Saclay, Orsay, France.

*Correspondence to Magdy Mahfouz (Magdy.mahfouz@kaust.edu.sa)

Additional File 1:

Figure S1: GEX1A and PB splicing modulators inhibit rice seed germination and primary root growth.

Figure S2: Conservation of SF3B1 in eukaryotes.

Figure S3: Domain focused directed evolution of SF3B1

Figure S4: Genotyping of *SGS1*

Figure S5: Generation of SF3B1 variants using domain focused CDE platform

Figure S6: Analysis of Seed progeny of SF3B1 mutant variants.

Figure S7: SGRs confer resistance to GEX1A treatment

Figure S8: SGRs primary root growth is not inhibited by GEX1A treatment.

Figure S9: SGRs primary root growth is not inhibited by PB treatment

Figure S10: The SF3B1 protein disorder plot and surface representation of 3D structure

Figure S11: The structural basis of SGRs resistance to PB

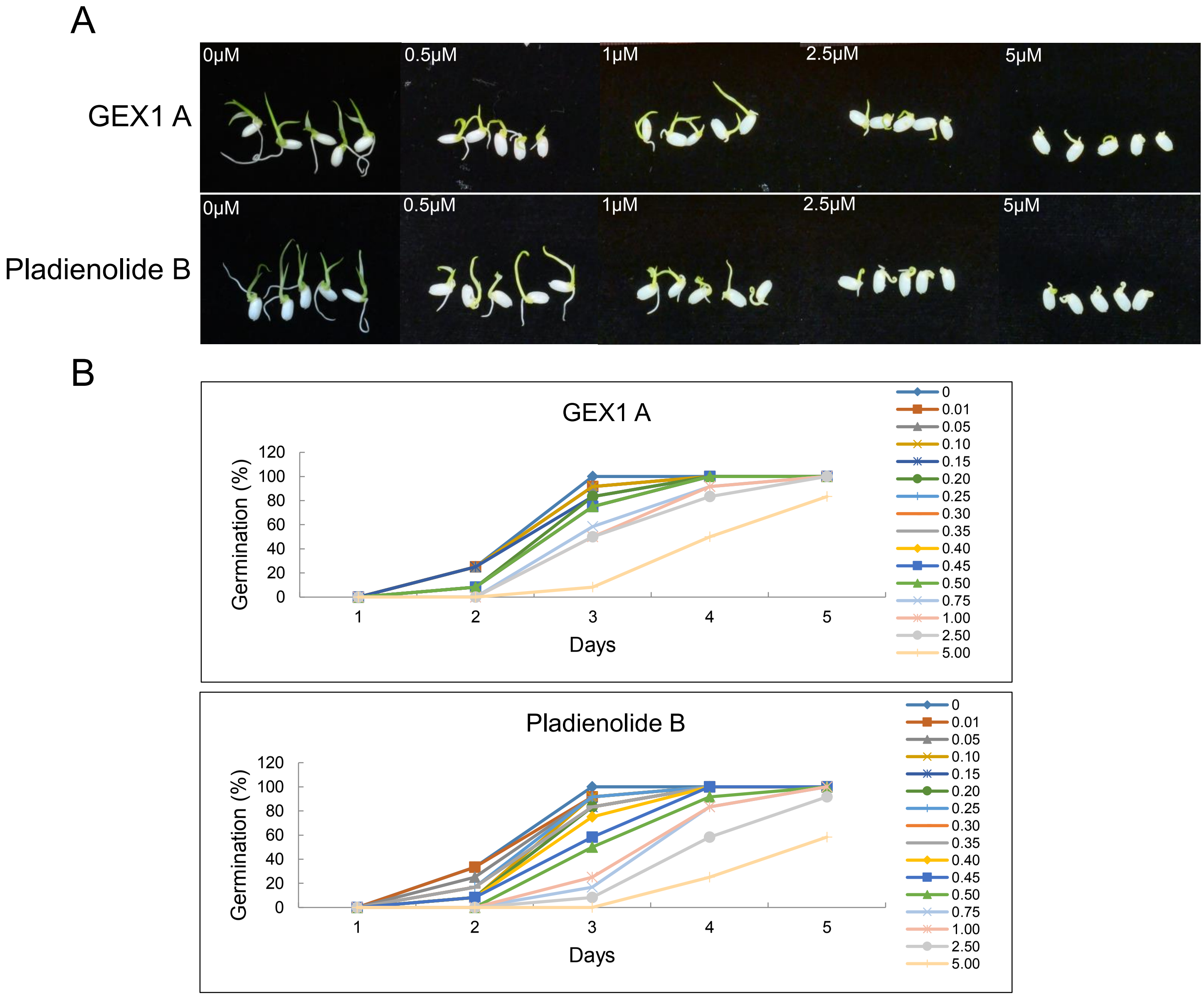
A
GEX1 A
Pladienolide B
0μM
0.5μM
1μM
2.5μM
5μM
0μM
0.5μM
1μM
2.5μM
5μM
B
GEX1 A
Germination (%)
120
100
80
60
40
20
0
1
2
3
4
5
Days
0
0.01
0.05
0.10
0.15
0.20
0.25
0.30
0.35
0.40
0.45
0.50
0.75
1.00
2.50
5.00
Pladienolide B
Germination (%)
120
100
80
60
40
20
0
1
2
3
4
5
Days
0
0.01
0.05
0.10
0.15
0.20
0.25
0.30
0.35
0.40
0.45
0.50
0.75
1.00
2.50
5.00

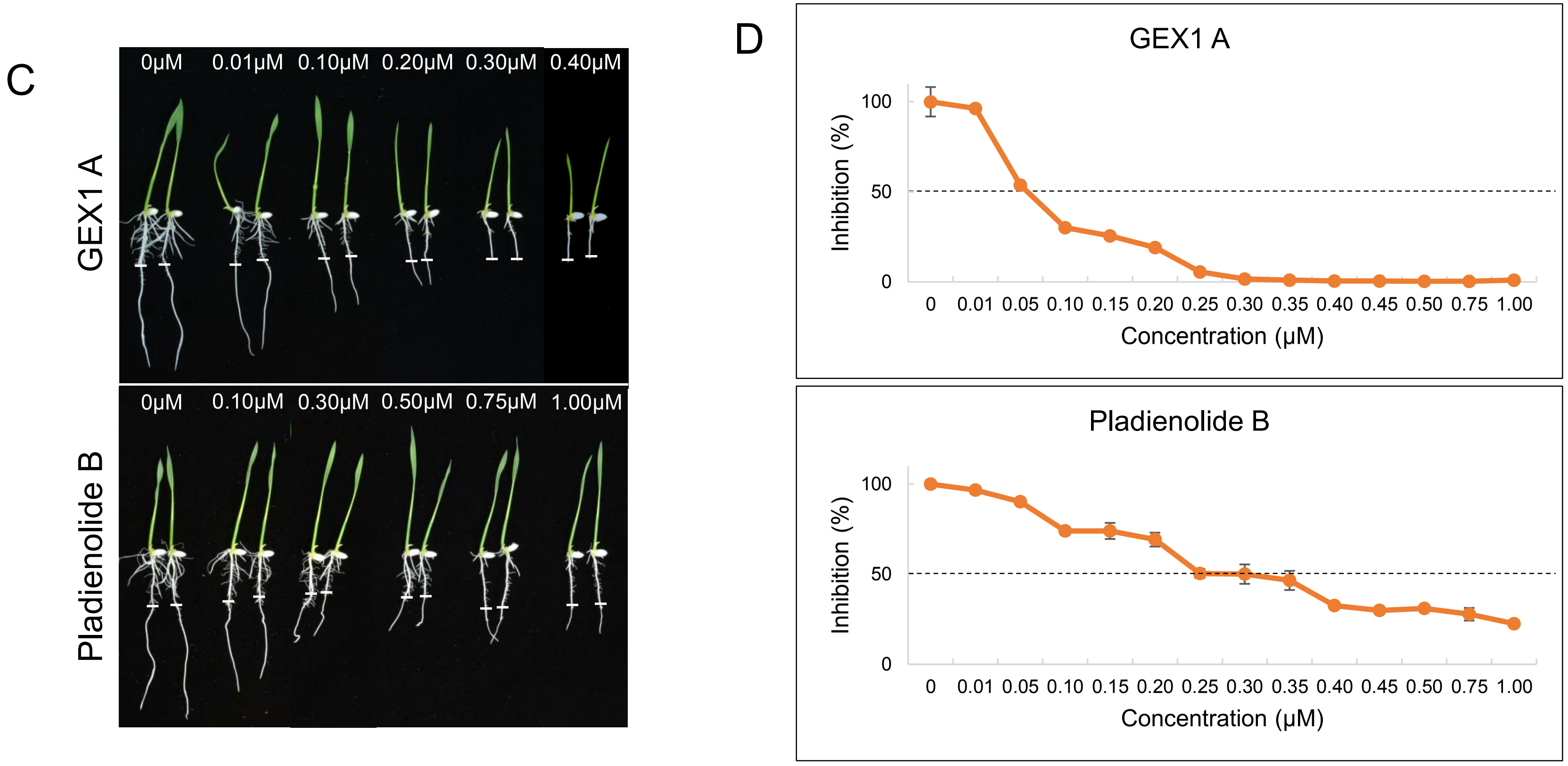

Figure S1: GEX1A and PB splicing modulators inhibit rice seed germination and primary root growth.

(A, B) Rice seeds were sterilized and germinated in dH_2O with different concentrations of PB and GEX1 A. Hypocotyl emergence was considered as germination. It was observed that the rice seed germination was affected in dose dependent manner. Germination was severely inhibited at 2.5 and 5µM concentrations of PB and GEX1A. (n=6) **(C, D)** Rice seedlings were germinated on ½ MS basal salt media plates for 3 days in vertical position. Seedlings of similar root size were transferred to ½ MS media plates supplemented with different concentrations of PB and GEX1 A. The primary root elongation was inhibited in dose dependent manner. (n=6)

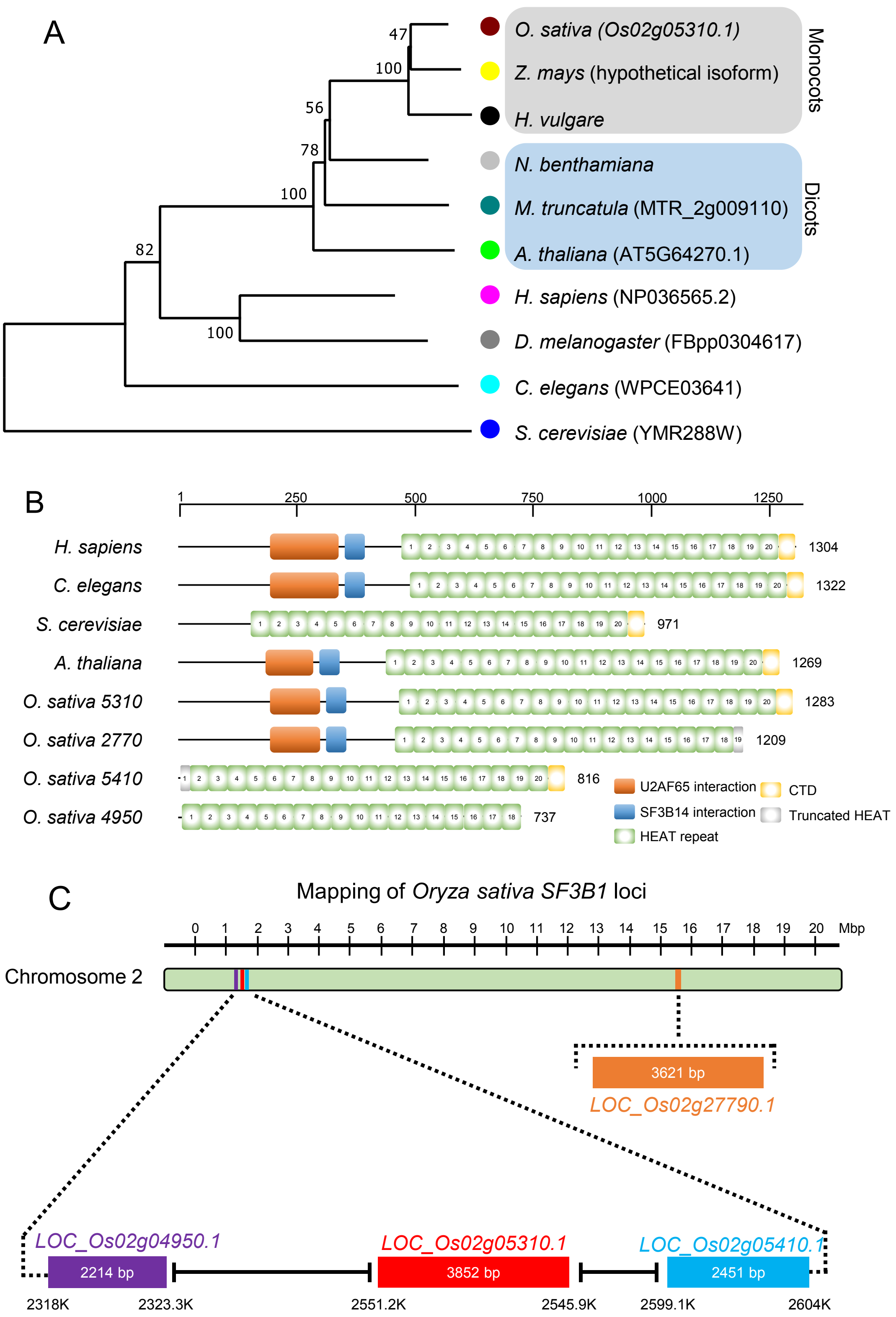

Figure S2: Conservation of SF3B1 in eukaryotes.
(A) Phylogenetic tree displays the evolutionary relationships among various species. **(B)** Domain structure of SF3B1 in different species. **(C)** Chromosomal locations of OsSF3B1 loci in rice.

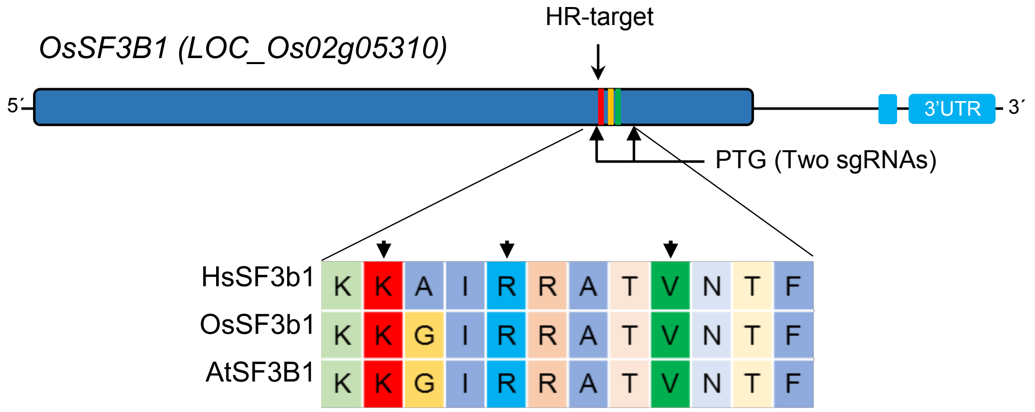

Figure S3: Domain focused directed evolution of SF3B1
HEAT repeat 15 is highly conserved among eukaryotes. Splicing modulators are known to bind this pocket. The arrows indicate the *H. sapiens* K1071, R1074 and V1078 are binding sites for different splicing modulators. This region was targeted by a single sgRNA (named HR (HEAT Repeat)-target) or with PTG fragment with two sgRNAs.

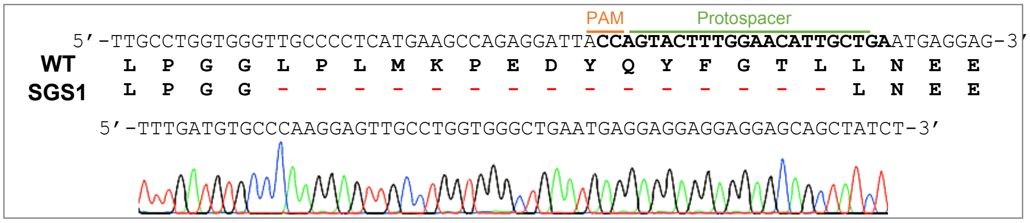

Figure S4: Genotyping of *SGS1*

The sensitive plant genotyped by Sanger sequencing and revealed in-frame deletions in *OsSF3B1* gene. This mutant was named as SGS1 (<u>S</u>F3B1 <u>G</u>EX1 <u>S</u>ensitive).

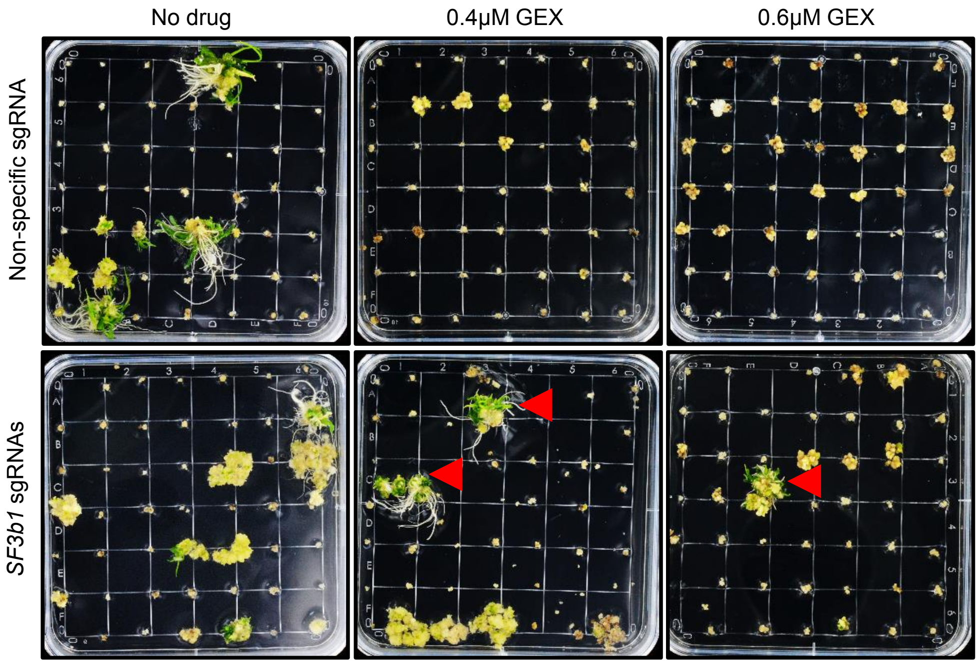

Figure S5: Generation of SF3B1 variants using domain focused CDE platform
Agrobacterium mediated transformation was conducted using sgRNA library targeting the SF3B1. After selection on hygromycin, regeneration was performed under selection pressure of GEX1A (0.4 µM or 0.6 µM). A non-specific sgRNA was transformed and used as GEX1A selection control. Regeneration was only observed in sgRNA library targeting SF3B1. Red arrows indicate the GEX1A-resistant shoots.

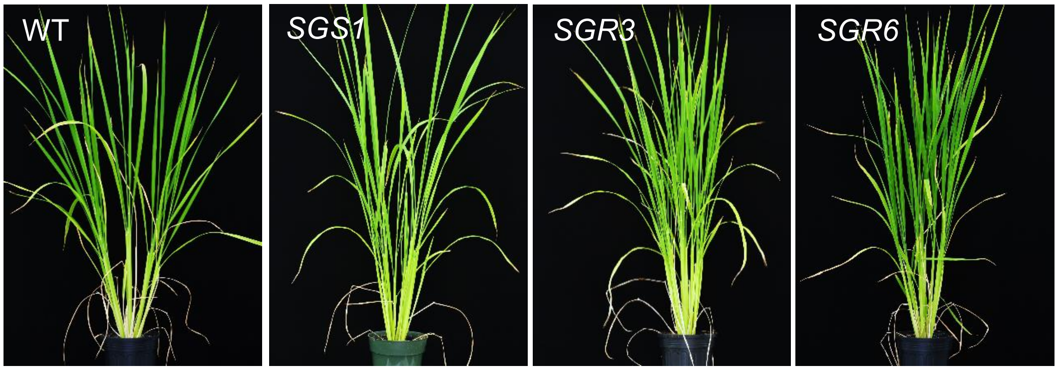

Figure S6: Analysis of Seed progeny of SF3B1 mutant variants.
SF3B1 variant mutants at 50-days after germination. No phenotypic differences among wild type and SF3B1 variants were observed.

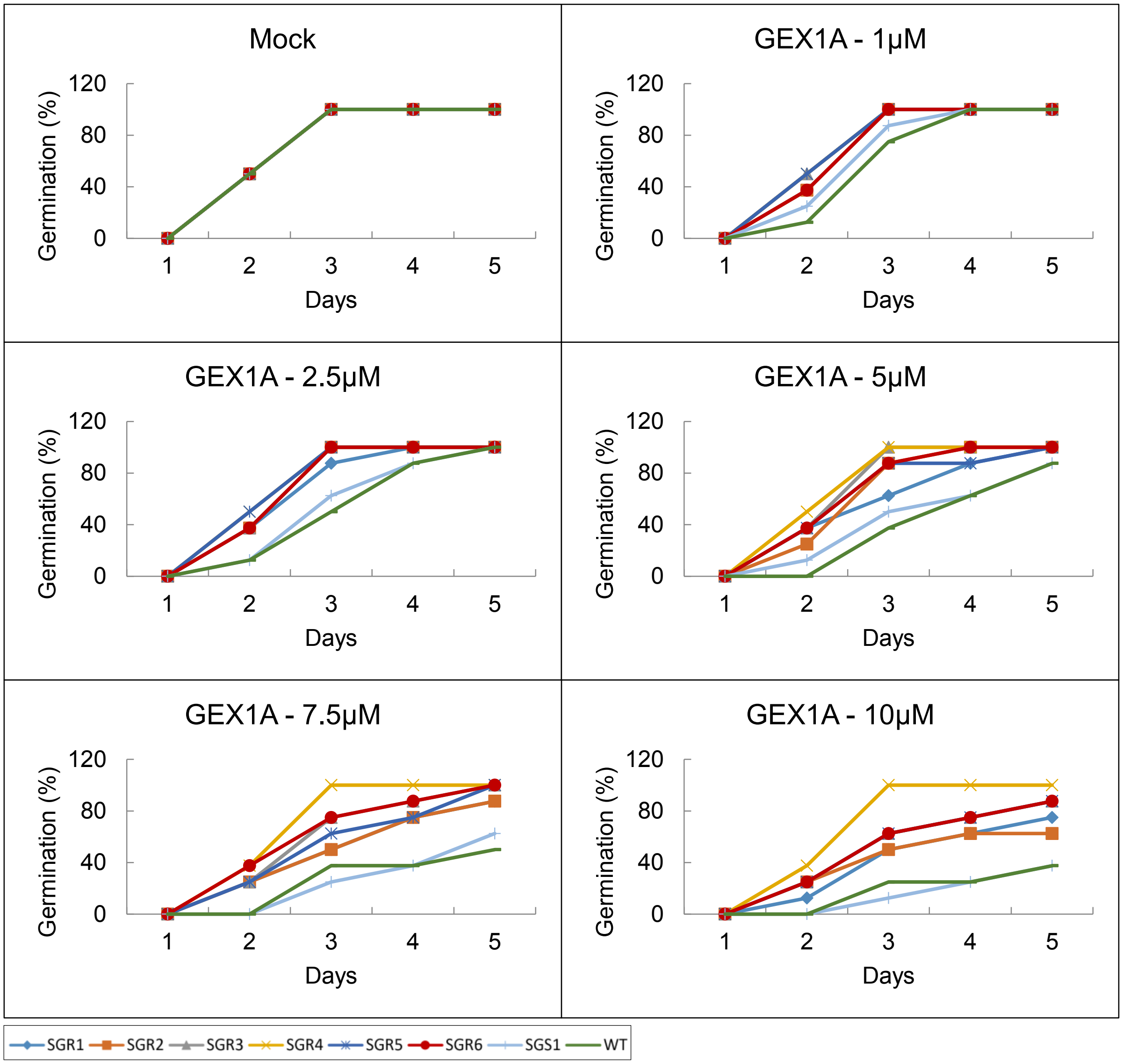

Figure S7: SGRs confer resistance to GEX1A treatment
Dose-response effects of GEX1A treatment on germination of WT, SGS, SGR1, SGR2, SGR3, SGR4, SGR5, and SGR6 seeds. Germination of wild type and SGS1 seeds was severely inhibited while germination of SGRs is less affected by 2.5 and 5μM concentrations of GEX1A. SGR4 germination is completely unaffected even at 10μM GEX1A concentration. (n=8)

A

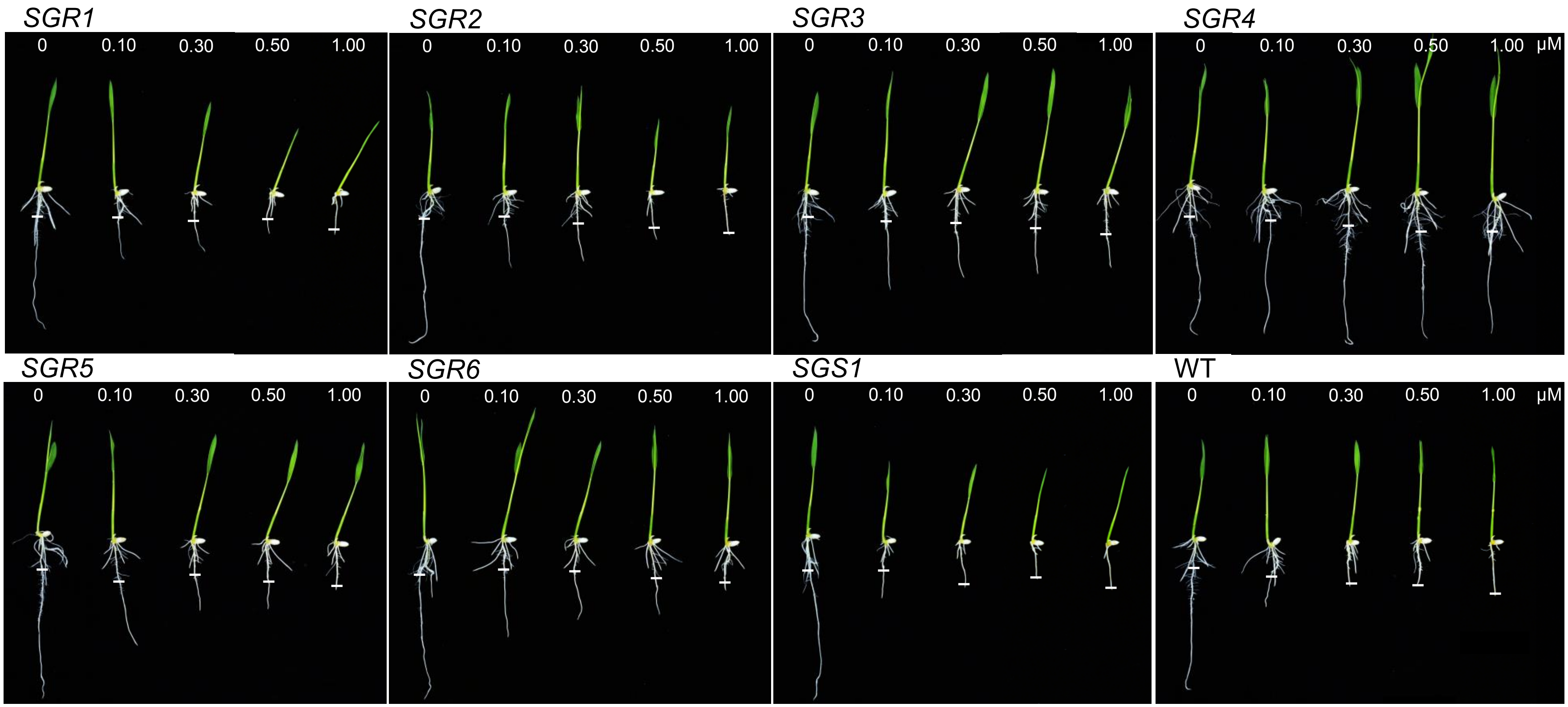
SGR1
0
0.10
0.30
0.50
1.00
SGR2
0
0.10
0.30
0.50
1.00
SGR3
0
0.10
0.30
0.50
1.00
SGR4
0
0.10
0.30
0.50
1.00
μM
SGR5
0
0.10
0.30
0.50
1.00
SGR6
0
0.10
0.30
0.50
1.00
SGS1
0
0.10
0.30
0.50
1.00
WT
0
0.10
0.30
0.50
1.00
μM

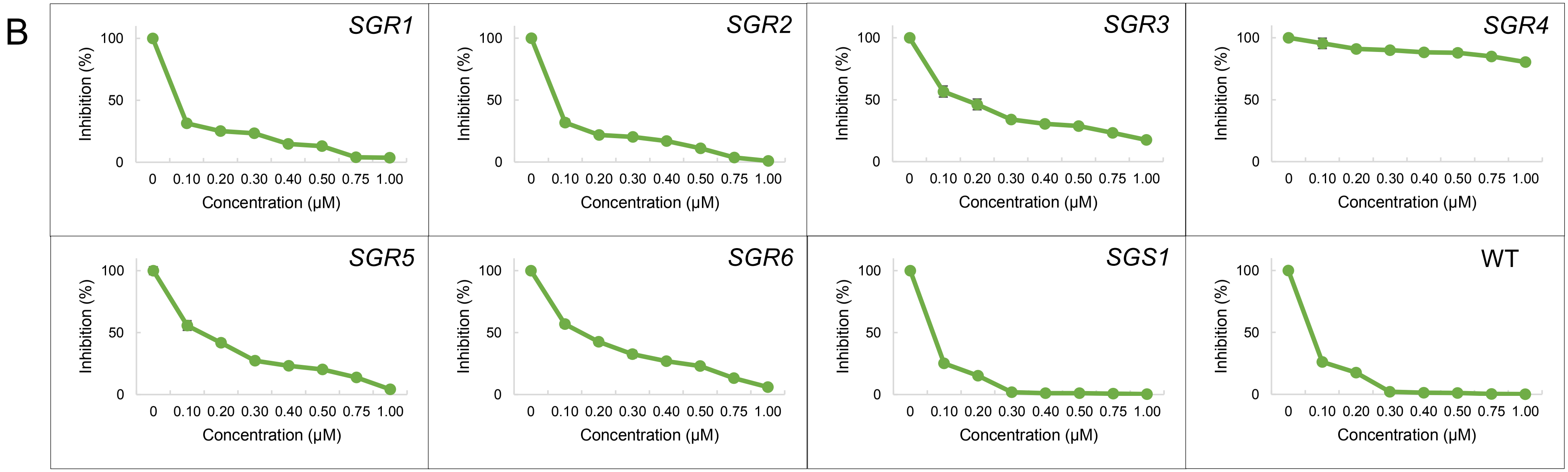

Figure S8: SGRs primary root growth is not inhibited by GEX1A treatment.
(A, B) Rice seedlings were germinated on ½ MS media plates for 3 days in vertical position. Seedlings of similar root size were transferred to ½MS media supplemented with different concentrations of GEX1A. The primary root elongation was inhibited in Wild type and SGS1 seedlings while SGRs are showing resistance to GEX1A. The primary root growth of SGR4 is not inhibited by GEX1A. (n=3)

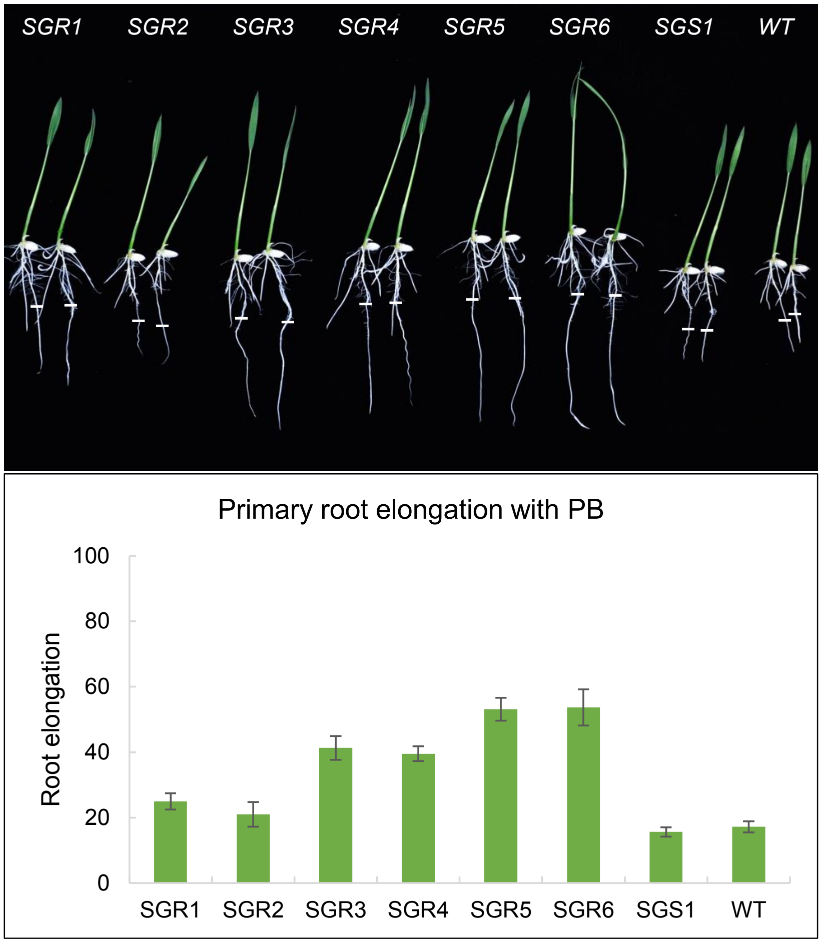

Figure S9: SGRs primary root growth is not inhibited by PB treatment
(A, B) Rice seedlings were germinated on ½ MS basal salt media plates for 3 days in vertical position. Seedlings of similar root size were transferred to ½ MS media plates supplemented with 1µM PB. SGR1 is partially resistant to PB. SGR2 are showing sensitivity to the drug. (n=3)

A
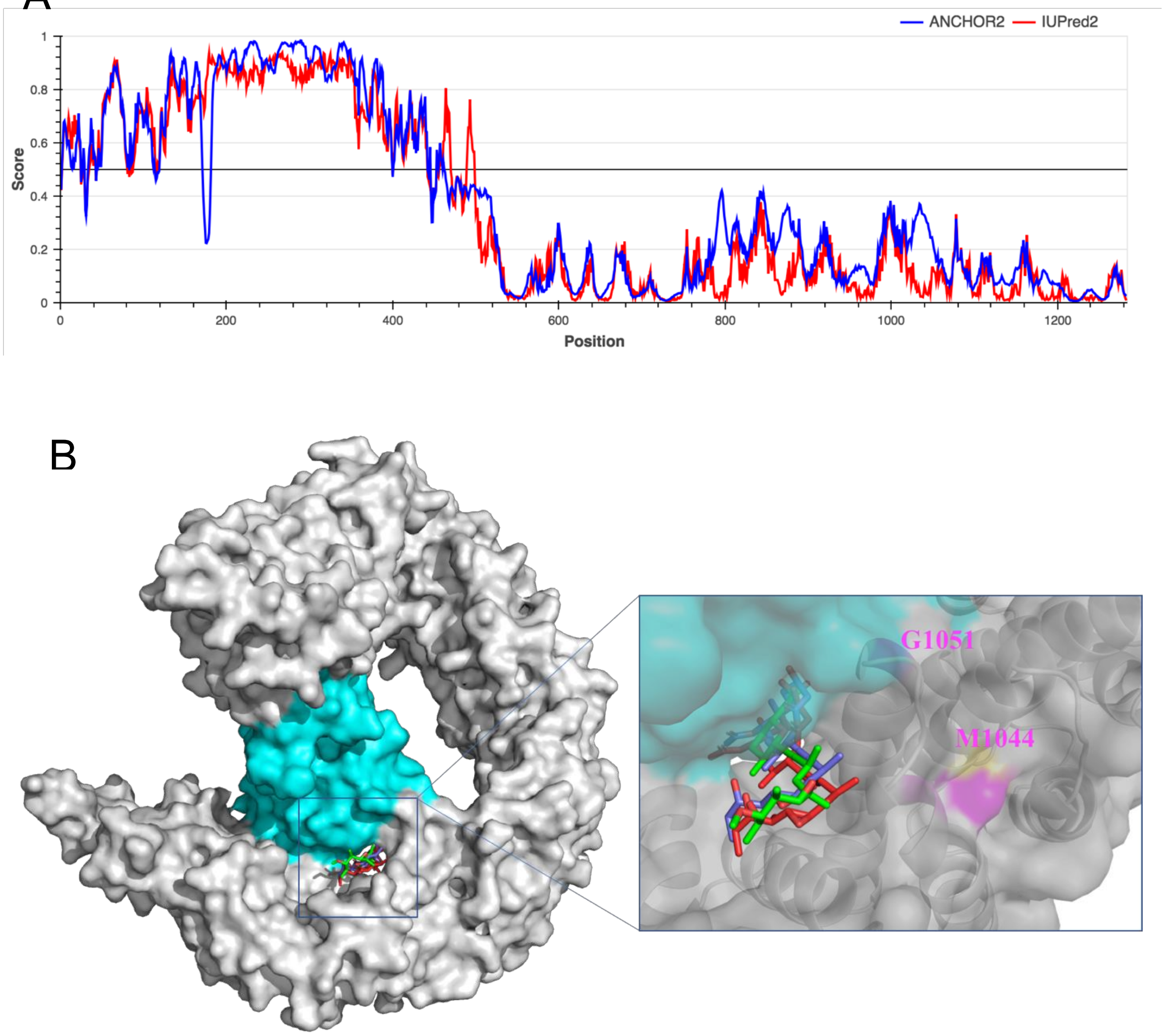

B

Figure S10: The SF3B1 protein disorder plot and surface representation of 3D structure **(A)** The protein disorder plot for OsSF3B1. Residues with values above 0.5 are predicted to be disordered. **(B)** Surface representation of the inferred 3D structure of the complex formed by OsSF3B1 (gray) and PHF5A (cyan). Docked small-molecule compounds are shown as stick models (GEX1A in green; PB in red and PD with the coordinated from crystal structure PDB id:6en4 in blue). The amino acids positions in the drug binding pocket differing between human and rice SF3B1 are shown and labelled in magenta.

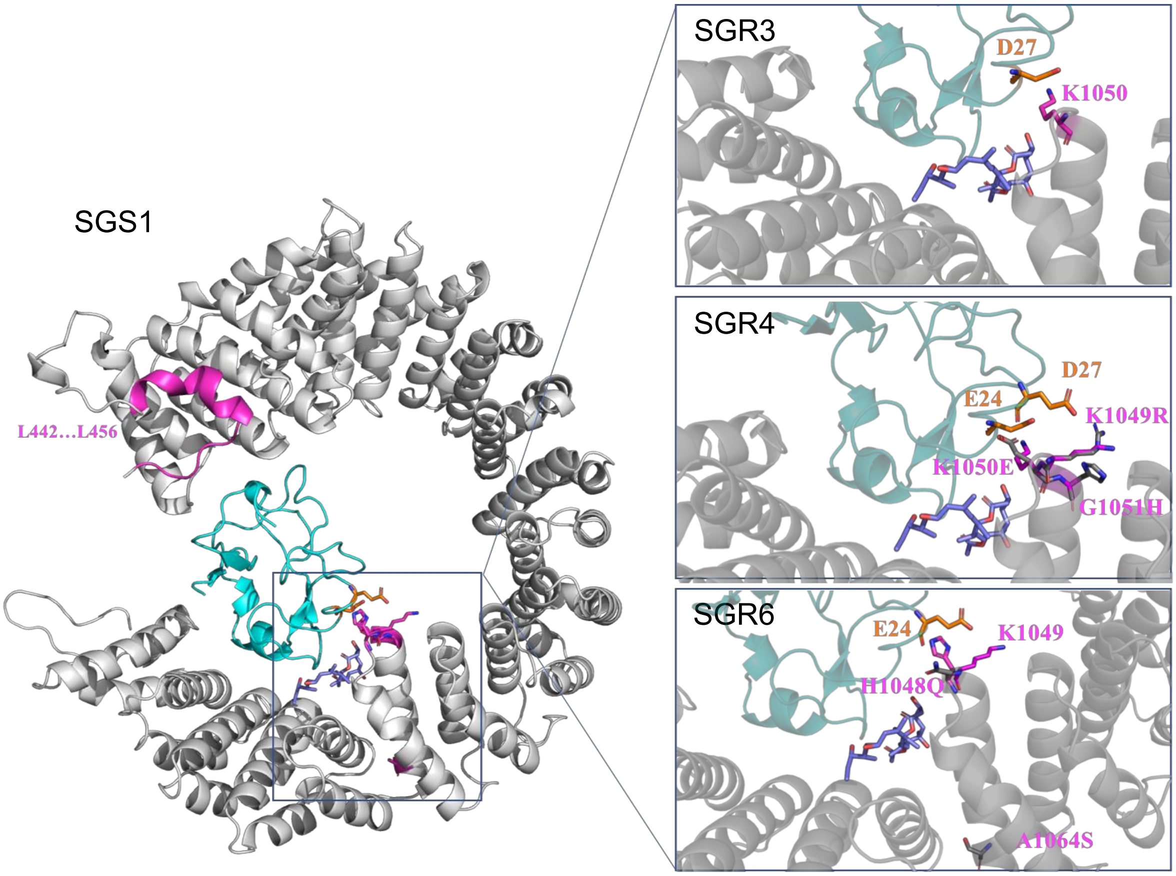

Figure S11: The structural basis of SGRs resistance to PB

The 3D model for OsSF3B1:PHF5A complex and PB with OsSF3B1 represented in gray cartoon; PHF5A represented in cyan cartoon and PB represented in blue sticks. Key residues on PHF5A are shown in orange sticks whereas residues for wild type are shown in magenta and mutations on OsSF3B1 are shown in gray sticks; both are marked respectively.
