# Supplementary material for: CRISPR directed evolution of the spliceosome for resistance to splicing inhibitors
Source: Genome Biol. 2019 Apr 30;20:73. doi: 10.1186/s13059-019-1680-9 (PMC6489355; doi:10.1186/s13059-019-1680-9)
Supplement: Supplementary file 2 — Table S1. Sequences of sgRNAs with PAM targeting SF3B1. Table S2. List of primers annealed to synthesis sgRNAs with BsaI compatible overhangs. Table S3. List of primers used for genotyping. Table S4. CDE platform for SF3B1 resistance to splicing inhibitor Table S5. List of primers used for intron retention analysis. (PDF 288 kb) [file 13059_2019_1680_MOESM2_ESM.pdf]

Additional file 2

CRISPR-directed evolution of the spliceosome for resistance to splicing inhibitors

Haroon Butt[1], Ayman Eid[1], Afaque A. Momin[2], Jeremie Bazin[3], Martin Crespi[3], Stefan T. Arold[2], and Magdy M. Mahfouz[1,*]

[1]Laboratory for Genome Engineering and Synthetic Biology, King Abdullah University of Science and Technology (KAUST), Thuwal, Saudi Arabia, [2]Division of Biological and Environmental Sciences and Engineering, Computational Bioscience Research Center, KAUST, Thuwal, Saudi Arabia, and [3]CNRS, INRA, Institute of Plant Sciences Paris-Saclay IPS2, Univ Paris Sud, Univ Evry, Univ Paris-Diderot, Sorbonne Paris-Cite, Universite Paris-Saclay, Orsay, France.

*Correspondence to Magdy Mahfouz (Magdy.mahfouz@kaust.edu.sa)

Additional File 2:

Table S1: Sequences of sgRNAs with PAM targeting SF3B1
Table S2: List of primers annealed to synthesis sgRNAs with *Bsa*I compatible overhangs
Table S3: List of primers used for genotyping
Table S4: CDE-platform for SF3B1 resistance to splicing inhibitor
Table S5: List of primers used for Intron retention analysis

Table S1: Sequences of sgRNAs with PAM targeting SF3B1

sgRNA	5´-sgRNA sequence-3´ (PAM is underlined)
1	GGAGCTCGCGCGGGCGCAGGAGG
2	GGAGCGCAAGAAGATGGAGGAGG
3	CCGCCGGCGCGCCCATGGCCGTC
4	CCTTCGACACCGATCTCTACGGC
5	CCGACCCCAACCGCTTCGCCGGC
6	CCTCCATCCCCGCCTCCGAGGAC
7	CTCCGAGGACGACGCGCCCGAGG
8	CCGAGGCGGCCGTCAACCCCGCC
9	CCCGCCGCCTCGCCTCCTACACC
10	CCGGCCACGCCGTCGCCGCCGCC
11	CGACATCCCCCGCGCCGCCGAGG
12	CCAAGAAGTCGCAGCGCATCATC
13	CCGCGAGGATGACTATCGCCGCC
14	GCTGGCCCGCATCATCTCGCCGG
15	CCGGAGCGCCATGATCCCTTCGC
16	CCACCCCGGACCCTTCCGTGCGG
17	CCGATGCCATGCGCGAGAATGAC
18	CCTGCAGAAGCAGAAGGAGCAGC
19	TGACATAGCTCAGAAGAAGAAGG
20	GGAGAAGGCCAAGGAGAAGAAGG
21	GCCGAAGCGCCGCAATAGATGGG
22	CAATAGATGGGATCAGTCGCAGG
23	GGTGATGCTTCTGCTGCTGCTGG
24	GGCGAAGACCTCCTCCGATTGGG
25	GATGCTCCTGATGCAACTCCTGG
26	GGGCGCTGGGATGCCACCCCTGG
27	CCCCTGGTCGTGTCGGGGATGCG
28	GTCGGTGAGAAGGAATCGTTGGG
29	CCGTCGGTGAGAAGGAATCGTTG
30	CCGATGCGGATGCAACACCTGCA
31	GCAGCTGGTGGCATTACTCCAGG
32	CCTTCTGGAGCTTGGGATGCTAC
33	CCTGGTGGGCTTGTCACGCCAAC
34	CCAACACCCAAGAAGCAGAGATC
35	CCTGCAAGTATGGGTAGTGCTAC
36	CCTGGAGGCACTGGTGCTGCGAC
37	CCTGCAGGTTACACTCCTGGTCC
38	CCATTTGGTGGTGACAACCTTGC
39	CCAACACCCGGCCAGATTGCTTC
40	CAGAGCAGTACCAGCTCTTGCGG
41	GAACAGGCCTCTTACGGATGAGG
42	GCTTGATACTATGTTCCCGCAGG
43	CCGCAGGAAGGGTACAAGATTCT
44	CCAGCTTCATACCAACCCATACG
45	CCAGCAAGAAAGCTGCTCGCTAC
46	ACCATTGTATGCTATTCCTGAGG
47	GCTATTCCTGAGGAGAATCGTGG
48	GTGCCCAAGGAGTTGCCTGGTGG
49	GTTGCCCCTCATGAAGCCAGAGG
50	CCAGTACTTTGGAACATTGCTGA
51	GCTATCTCCAGAAGAGCAGAAGG
52	GATCATGAAGCTCCTGCTCAAGG
53	CCACAGCGGAAGACAGCACTTCG

54	GCTCATGCAGCCAACACTTGAGG
55	TGACAGGGTACTGTATAAGCTGG
56	CCGTCCGTTTGTGCATAAGATTC
57	GACTACTATGCTCGTGTTGAGGG
58	CCGGGAGATTATCTCAAATCTTA
59	TCAAATCTTAGCAAAGCTGCTGG
60	CCGCCATGAGACCAGATATTGAT
61	CACTGCTAGAGCATTTAGCGTGG
62	TAGCGTGGTGGCTTCTGCTTTGG
63	TCCTGGCAAGCTCGGCACACTGG
64	CCTGGCAAGCTCGGCACACTGGT
65	CAGCAGATTGCTATTCTTATGGG
66	CCTAGTTGAGATCATTGAGCATG
67	GTGACGAGAACCAGAAAGTGCGG
68	CCAGAAAGTGCGGACAATCACTG
69	AAGGGTATCAGATCTCACCGTGG
70	CCGTGGAAAGGTTCTTGCTGCCT
71	GCTGCCTTCTTGAAAGCTATTGG
72	CCTCTTATGGATGCGCTGTATGC
73	CCTGATTAGGGAGTTCCAGTCAC
74	GATGAAGAAGATTGTCCTGAAGG
75	GTGTAGAGGCTGATTATATCCGG
76	GGAGAATGGCTCTGGACCGCAGG
77	GTAGAGATGGCAAATAAGGTTGG
78	TATTGTTGGGAGGATTGTTGAGG
79	GCCTTACAGGAGAATGGTGATGG
80	GGTGATGGAAACAATTGAGAAGG
81	TTGGAGGAGCTGCTTATTGATGG
82	CCTTACCTTCCTCAGATTTGTGG
83	AAGCTGCTGATCTGATCTCGAGG
84	GTGTCAGGAGGAGCAGCTTATGG
85	GAAGAGTACCCTGAGGTGTTGGG
86	CCGCCAATCAAGGATCTTCTTCC
87	GAATAGGCATGAGAAGGTCCAGG
88	GAGAACTGCATTGATCTAGTTGG
89	GTTGGTAGGATTGCTGATCGTGG
90	TGCCTGCTAGGGAGTGGATGAGG
91	AATGTTGAAGGCTCACAAGAAGG
92 (HR-target)	ATGTTGAAGGCTCACAAGAAGGG
93	AGGCTCACAAGAAGGGTATTAGA
94	CTCACAAGAAGGGTATTAGAAGA
95	GGTTATATTGCGAAGGCTATTGG
96	GTTGAATAACTTGAAGGTGCAGG
97	CCGTGTCTGCACAACTGTCGCAA
98	CCTGATGAATGAGTACCGAGTCC
99	GTATATTGGTGAGATGGGCAAGG
100	CCTTGCTTGAAGATGCTCTAATG
101	GCTGTGAAGCACATGGCTCTTGG
102	TGTTGCTGGCTTGGGCTGTGAGG
103	GCTGTCATGGAGGCGATTGAGGG
104	GAGGGGATGAGAGTTGCTCTTGG
105	CCAGCTGTGATCTTGAATTACTG
106	CCATCCAGCAAGGAAAGTACGTG
107	TCCTGCACTGGATGACGATGGGG

108	TATCTACAGCCGTCCAGAGCTGG
109	GATGAGACTCCTGCAAGTATGGG
110	CCAGAAGAGCAGAAGGAGAGGAA
111	AAAGCAAGGGAATTTGGTGCTGG
112	CCATTGTTCAACAAGATTCTACC
113	CCAGATATTGATAACATTGATGA
114	ATAACATTGATGAGTATGTGAGA
115	CCACTGCTAGAGCATTTAGCGTG
116	CCAGTCACCAGATGAAGAGATGA
117	CCAGATGAAGAGATGAAGAAGAT
118	CCGGCAAACTGCTGCCTCTGCTG
119	CACATTTGGTTATATTGCGAAGG

Table S2: List of primers annealed to synthesis sgRNAs with *Bsa*I compatible overhangs

Name of the primer	Sequence (5' - 3')
F1	GGCAGGAGCTCGCGCGGGCGCAGG
F2	GGCAGGAGCGCAAGAAGATGGAGG
F3	GGCAGACGGCCATGGGCGCGCCGG

F4	GGCAGCCGTAGAGATCGGTGTCGA
F5	GGCAGCCGGCGAAGCGGTTGGGGT
F6	GGCAGTCCTCGGAGGCGGGGATGG
F7	GGCACTCCGAGGACGACGCGCCCG
F8	GGCAGGCGGGGTTGACGGCCGCCT
F9	GGCAGGTGTAGGAGGCGAGGCGGC
F10	GGCAGGCGGCGGCGACGGCGTGGC
F11	GGCACGACATCCCCCGCGCCGCCG
F12	GGCAGATGATGCGCTGCGACTTCT
F13	GGCAGGCGGCGATAGTCATCCTCG
F14	GGCAGCTGGCCCGCATCATCTCGC
F15	GGCAGCGAAGGGATCATGGCGCTC
F16	GGCACCACCCCGGACCCTTCCGTG
F17	GGCAGTCATTCTCGCGCATGGCAT
F18	GGCAGCTGCTCCTTCTGCTTCTGC
F19	GGCATGACATAGCTCAGAAGAAGA
F20	GGCAGGAGAAGGCCAAGGAGAAGA
F21	GGCAGCCGAAGCGCCGCAATAGAT
F22	GGCACAATAGATGGGATCAGTCGC
F23	GGCAGGTGATGCTTCTGCTGCTGC
F24	GGCAGGCGAAGACCTCCTCCGATT
F25	GGCAGATGCTCCTGATGCAACTCC
F26	GGCAGGGCGCTGGGATGCCACCCC
F27	GGCACGCATCCCCGACACGACCAG
F28	GGCAGTCGGTGAGAAGGAATCGTT
F29	GGCACAACGATTCCTTCTCACCGA
F30	GGCATGCAGGTGTTGCATCCGCAT
F31	GGCAGCAGCTGGTGGCATTACTCC
F32	GGCAGTAGCATCCCAAGCTCCAGA
F33	GGCAGTTGGCGTGACAAGCCCACC
F34	GGCAGATCTCTGCTTCTTGGGTGT
F35	GGCAGTAGCACTACCCATACTTGC
F36	GGCAGTCGCAGCACCAGTGCCTCC
F37	GGCAGGACCAGGAGTGTAACCTGC
F38	GGCAGCAAGGTTGTCACCACCAAA
F39	GGCAGAAGCAATCTGGCCGGGTGT
F40	GGCACAGAGCAGTACCAGCTCTTG
F41	GGCAGAACAGGCCTCTTACGGATG
F42	GGCAGCTTGATACTATGTTCCCGC
F43	GGCAAGAATCTTGTACCCTTCCTG
F44	GGCACGTATGGGTTGGTATGAAGC
F45	GGCAGTAGCGAGCAGCTTTCTTGC
F46	GGCAACCATTGTATGCTATTCCTG
F47	GGCAGCTATTCCTGAGGAGAATCG

F48	GGCAGTGCCCAAGGAGTTGCCTGG
F49	GGCAGTTGCCCCTCATGAAGCCAG
F50	GGCATCAGCAATGTTCCAAAGTAC
F51	GGCAGCTATCTCCAGAAGAGCAGA
F52	GGCAGATCATGAAGCTCCTGCTCA
F53	GGCACGAAGTGCTGTCTTCCGCTG
F54	GGCAGCTCATGCAGCCAACACTTG
F55	GGCATGACAGGGTACTGTATAAGC
F56	GGCAGAATCTTATGCACAAACGGA
F57	GGCAGACTACTATGCTCGTGTTGA
F58	GGCATAAGATTTGAGATAATCTCC
F59	GGCATCAAATCTTAGCAAAGCTGC
F60	GGCAATCAATATCTGGTCTCATGG
F61	GGCACACTGCTAGAGCATTTAGCG
F62	GGCATAGCGTGGTGGCTTCTGCTT
F63	GGCATCCTGGCAAGCTCGGCACAC
F64	GGCAACCAGTGTGCCGAGCTTGCC
F65	GGCACAGCAGATTGCTATTCTTAT
F66	GGCACATGCTCAATGATCTCAACT
F67	GGCAGTGACGAGAACCAGAAAGTG
F68	GGCACAGTGATTGTCCGCACTTTC
F69	GGCAAAGGGTATCAGATCTCACCG
F70	GGCAAGGCAGCAAGAACCTTTCCA
F71	GGCAGCTGCCTTCTTGAAAGCTAT
F72	GGCAGCATACAGCGCATCCATAAG
F73	GGCAGTGACTGGAACTCCCTAATC
F74	GGCAGATGAAGAAGATTGTCCTGA
F75	GGCAGTGTAGAGGCTGATTATATC
F76	GGCAGGAGAATGGCTCTGGACCGC
F77	GGCAGTAGAGATGGCAAATAAGGT
F78	GGCATATTGTTGGGAGGATTGTTG
F79	GGCAGCCTTACAGGAGAATGGTGA
F80	GGCAGGTGATGGAAACAATTGAGA
F81	GGCATTGGAGGAGCTGCTTATTGA
F82	GGCACCACAAATCTGAGGAAGGTA
F83	GGCAAAGCTGCTGATCTGATCTCG
F84	GGCAGTGTCAGGAGGAGCAGCTTA
F85	GGCAGAAGAGTACCCTGAGGTGTT
F86	GGCAGGAAGAAGATCCTTGATTGG
F87	GGCAGAATAGGCATGAGAAGGTCC
F88	GGCAGAGAACTGCATTGATCTAGT
F89	GGCAGTTGGTAGGATTGCTGATCG
F90	GGCATGCCTGCTAGGGAGTGGATG
F91	GGCAAATGTTGAAGGCTCACAAGA

F92 (HR-target)	GGCAATGTTGAAGGCTCACAAGAA
F93	GGCAAGGCTCACAAGAAGGGTATT
F94	GGCACTCACAAGAAGGGTATTAGA
F95	GGCAGGTTATATTGCGAAGGCTAT
F96	GGCAGTTGAATAACTTGAAGGTGC
F97	GGCATTGCGACAGTTGTGCAGACA
F98	GGCAGGACTCGGTACTCATTCATC
F99	GGCAGTATATTGGTGAGATGGGCA
F100	GGCACATTAGAGCATCTTCAAGCA
F101	GGCAGCTGTGAAGCACATGGCTCT
F102	GGCATGTTGCTGGCTTGGGCTGTG
F103	GGCAGCTGTCATGGAGGCGATTGA
F104	GGCAGAGGGGATGAGAGTTGCTCT
F105	GGCACAGTAATTCAAGATCACAGC
F106	GGCACACGTACTTTCCTTGCTGGA
F107	GGCATCCTGCACTGGATGACGATG
F108	GGCATATCTACAGCCGTCCAGAGC
F109	GGCAGATGAGACTCCTGCAAGTAT
F110	GGCATTCCTCTCCTTCTGCTCTTC
F111	GGCAAAAGCAAGGGAATTTGGTGC
F112	GGCAGGTAGAATCTTGTTGAACAA
F113	GGCATCATCAATGTTATCAATATC
F114	GGCAATAACATTGATGAGTATGTG
F115	GGCACACGCTAAATGCTCTAGCAG
F116	GGCATCATCTCTTCATCTGGTGAC
F117	GGCAATCTTCTTCATCTCTTCATC
F118	GGCACAGCAGAGGCAGCAGTTTGC
F119	GGCACACATTTGGTTATATTGCGA
R1	AAACCCTGCGCCCGCGCGAGCTCC
R2	AAACCCTCCATCTTCTTGCGCTCC
R3	AAACCCGGCGCGCCCATGGCCGTC
R4	AAACTCGACACCGATCTCTACGGC
R5	AAACACCCCAACCGCTTCGCCGGC
R6	AAACCCATCCCCGCCTCCGAGGAC
R7	AAACCGGGCGCGTCGTCCTCGGAG
R8	AAACAGGCGGCCGTCAACCCCGCC
R9	AAACGCCGCCTCGCCTCCTACACC
R10	AAACGCCACGCCGTCGCCGCCGCC
R11	AAACCGGCGGCGCGGGGGATGTCG
R12	AAACAGAAGTCGCAGCGCATCATC
R13	AAACCGAGGATGACTATCGCCGCC
R14	AAACGCGAGATGATGCGGGCCAGC
R15	AAACGAGCGCCATGATCCCTTCGC
R16	AAACCACGGAAGGGTCCGGGGTGG

R17	AAACATGCCATGCGCGAGAATGAC
R18	AAACGCAGAAGCAGAAGGAGCAGC
R19	AAACTCTTCTTCTGAGCTATGTCA
R20	AAACTCTTCTCCTTGGCCTTCTCC
R21	AAACATCTATTGCGGCGCTTCGGC
R22	AAACGCGACTGATCCCATCTATTG
R23	AAACGCAGCAGCAGAAGCATCACC
R24	AAACAATCGGAGGAGGTCTTCGCC
R25	AAACGGAGTTGCATCAGGAGCATC
R26	AAACGGGGTGGCATCCCAGCGCCC
R27	AAACCTGGTCGTGTCGGGGATGCG
R28	AAACAACGATTCCTTCTCACCGAC
R29	AAACTCGGTGAGAAGGAATCGTTG
R30	AAACATGCGGATGCAACACCTGCA
R31	AAACGGAGTAATGCCACCAGCTGC
R32	AAACTCTGGAGCTTGGGATGCTAC
R33	AAACGGTGGGCTTGTCACGCCAAC
R34	AAACACACCCAAGAAGCAGAGATC
R35	AAACGCAAGTATGGGTAGTGCTAC
R36	AAACGGAGGCACTGGTGCTGCGAC
R37	AAACGCAGGTTACACTCCTGGTCC
R38	AAACTTTGGTGGTGACAACCTTGC
R39	AAACACACCCGGCCAGATTGCTTC
R40	AAACCAAGAGCTGGTACTGCTCTG
R41	AAACCATCCGTAAGAGGCCTGTTC
R42	AAACGCGGGAACATAGTATCAAGC
R43	AAACCAGGAAGGGTACAAGATTCT
R44	AAACGCTTCATACCAACCCATACG
R45	AAACGCAAGAAAGCTGCTCGCTAC
R46	AAACCAGGAATAGCATACAATGGT
R47	AAACCGATTCTCCTCAGGAATAGC
R48	AAACCCAGGCAACTCCTTGGGCAC
R49	AAACCTGGCTTCATGAGGGGCAAC
R50	AAACGTACTTTGGAACATTGCTGA
R51	AAACTCTGCTCTTCTGGAGATAGC
R52	AAACTGAGCAGGAGCTTCATGATC
R53	AAACCAGCGGAAGACAGCACTTCG
R54	AAACCAAGTGTTGGCTGCATGAGC
R55	AAACGCTTATACAGTACCCTGTCA
R56	AAACTCCGTTTGTGCATAAGATTC
R57	AAACTCAACACGAGCATAGTAGTC
R58	AAACGGAGATTATCTCAAATCTTA
R59	AAACGCAGCTTTGCTAAGATTTGA
R60	AAACCCATGAGACCAGATATTGAT

R61	AAACCGCTAAATGCTCTAGCAGTG
R62	AAACAAGCAGAAGCCACCACGCTA
R63	AAACGTGTGCCGAGCTTGCCAGGA
R64	AAACGGCAAGCTCGGCACACTGGT
R65	AAACATAAGAATAGCAATCTGCTG
R66	AAACAGTTGAGATCATTGAGCATG
R67	AAACCACTTTCTGGTTCTCGTCAC
R68	AAACGAAAGTGCGGACAATCACTG
R69	AAACCGGTGAGATCTGATACCCTT
R70	AAACTGGAAAGGTTCTTGCTGCCT
R71	AAACATAGCTTTCAAGAAGGCAGC
R72	AAACCTTATGGATGCGCTGTATGC
R73	AAACGATTAGGGAGTTCCAGTCAC
R74	AAACTCAGGACAATCTTCTTCATC
R75	AAACGATATAATCAGCCTCTACAC
R76	AAACGCGGTCCAGAGCCATTCTCC
R77	AAACACCTTATTTGCCATCTCTAC
R78	AAACCAACAATCCTCCCAACAATA
R79	AAACTCACCATTCTCCTGTAAGGC
R80	AAACTCTCAATTGTTTCCATCACC
R81	AAACTCAATAAGCAGCTCCTCCAA
R82	AAACTACCTTCCTCAGATTTGTGG
R83	AAACCGAGATCAGATCAGCAGCTT
R84	AAACTAAGCTGCTCCTCCTGACAC
R85	AAACAACACCTCAGGGTACTCTTC
R86	AAACCCAATCAAGGATCTTCTTCC
R87	AAACGGACCTTCTCATGCCTATTC
R88	AAACACTAGATCAATGCAGTTCTC
R89	AAACCGATCAGCAATCCTACCAAC
R90	AAACCATCCACTCCCTAGCAGGCA
R91	AAACTCTTGTGAGCCTTCAACATT
R92 (HR-target)	AAACTTCTTGTGAGCCTTCAACAT
R93	AAACAATACCCTTCTTGTGAGCCT
R94	AAACTCTAATACCCTTCTTGTGAG
R95	AAACATAGCCTTCGCAATATAACC
R96	AAACGCACCTTCAAGTTATTCAAC
R97	AAACTGTCTGCACAACTGTCGCAA
R98	AAACGATGAATGAGTACCGAGTCC
R99	AAACTGCCCATCTCACCAATATAC
R100	AAACTGCTTGAAGATGCTCTAATG
R101	AAACAGAGCCATGTGCTTCACAGC
R102	AAACCACAGCCCAAGCCAGCAACA
R103	AAACTCAATCGCCTCCATGACAGC
R104	AAACAGAGCAACTCTCATCCCCTC

R105	AAACGCTGTGATCTTGAATTACTG
R106	AAACTCCAGCAAGGAAAGTACGTG
R107	AAACCATCGTCATCCAGTGCAGGA
R108	AAACGCTCTGGACGGCTGTAGATA
R109	AAACATACTTGCAGGAGTCTCATC
R110	AAACGAAGAGCAGAAGGAGAGGAA
R111	AAACGCACCAAATTCCCTTGCTTT
R112	AAACTTGTTCAACAAGATTCTACC
R113	AAACGATATTGATAACATTGATGA
R114	AAACCACATACTCATCAATGTTAT
R115	AAACCTGCTAGAGCATTTAGCGTG
R116	AAACGTCACCAGATGAAGAGATGA
R117	AAACGATGAAGAGATGAAGAAGAT
R118	AAACGCAAACTGCTGCCTCTGCTG
R119	AAACTCGCAATATAACCAAATGTG

Table S3: List of primers used for genotyping

Name of the primer	Sequence (5' - 3')
F1	CTAACCGCCTCTCCCCCCGCC
F2	AAGAAGATGGAGGAGGCCCTT
F3	CTTCCGTGCGGACCTATGCC
F4	AAGGAGAAGAAGGCCGTGCCC
F5	ACCGTCGGTGAGAAGGAATCGT

F6	AGAAAGCTGCTCGCTACACCA
F7	GCGGAAGACAGCACTTCGACAA
F8	CAAAGCTGCTGGTCTTGCTACC
F9	CCACATCTCAAGTCCCTAGTT
F10	GATGGAAACAATTGAGAAGGTT
F11	ACAAGCTGCTGATCTGATCTCG
F12	GCAAAACCGTGTCTGCACA
F13	TTGCTTAACTATGTCTGGCCA
R1	CAACGGCAACAAAGTAAACATT
R2	CCACCGAGTTCAAGCTTCACAG
R3	CATAGAATTCTAACATATCAATGTA
R4	CAATAATATATGCCACAAAATTTAAGC
R5	TCATCAGGGCAGGCAGAACC
R6	ATAAGCTGCTCCTCCTGACAC
R7	TGAAGCACCCAAATTAGCCACA
R8	GTCTCATGGCGGCAATCATG
R9	GGTAATCCTCTGGCTTCATGAG
R10	ACGATTCCTTCTCACCGACGGT
R11	CTGCCACTGGCTGCTGCTCG
R12	CTTCCTTCTTCTTCTGAGCTATG
T-DNA-F	CACAGGAAACAGCTATGAC
T-DNA-R	CGCGCTAAAAACGGACTAG

Table S4: CDE-platform for SF3B1 resistance to splicing inhibitor

<table>
<tr><th></th><th colspan="3">CRISPR-directed evolution of SF3B1 via targeted sgRNA library</th><th colspan="5">Directed evolution of SF3B1 via domain-specific sgRNAs</th></tr>
<tr><td>No. of transformation</td><td colspan="3">2</td><td colspan="5">1 (One for each sgRNA)</td></tr>
<tr><td rowspan="2">No. of sgRNAs</td><td colspan="3" rowspan="2">119 (in an sgRNA libarary transformed to Agrobacterium)</td><td colspan="5">3</td></tr>
<tr><td colspan="3">sgRNA-92</td><td>sgRNA-119</td><td>PTG</td></tr>
<tr><td>No. of calli transformed</td><td colspan="3">~15,000</td><td colspan="3">1000</td><td>1000</td><td>1000</td></tr>
<tr><td>No. of shoots/plants recovered on GEX1A supplemented media</td><td colspan="3">21 seedlings from 0.4µM</td><td colspan="2">8 seedlings from 0.4µM</td><td>4 seedlings from 0.6µM</td><td>No seedlings</td><td>5 seedlings from 0.4µM</td></tr>
<tr><td rowspan="2">No. of plants showing SF3B1 editing</td><td>SGR1</td><td>SGR2</td><td>SGR3</td><td>SGR3</td><td>SGR5</td><td>SGR4</td><td rowspan="2">—</td><td>SGR6</td></tr>
<tr><td>2</td><td>2</td><td>3</td><td>3</td><td>2</td><td>2</td><td>2</td></tr>
<tr><td rowspan="2">Heritability of GEX1A resistance in seed progeny (T1)</td><td>SGR1</td><td>SGR2</td><td>SGR3</td><td>SGR3</td><td>SGR5</td><td>SGR4</td><td rowspan="2">___</td><td>SGR6</td></tr>
<tr><td>2</td><td>2</td><td>3</td><td>3</td><td>2</td><td>2</td><td>2</td></tr>
</table>

*Please note that all false positives exhibit no SF3B1 editing and their seed progeny is sensitive to GEX1A

**Please note that one set of transformation was conducted without GEX1A selection for targeted mutagenesis of SF3B1 and no functional knock out mutant could be recovered from these (except one in-frame mutant *SGS1*).

Table S5: List of primers used for Intron retention analysis

Name of the primer	Sequence (5' - 3')
03g55590_F	GCTACTCCAAAGCAAATCAGGGAG
03g55590_R	CGGACATAGCCTTCCGACTTG
04g37790_F	GTCTTCTAATTCGGTTCCCTGACACG
04g37790_R	GGGGACGGCTTGGAATCCTTG
04g57440_F	GCTAAGGTTGTGAGCATCGATGG
04g57440_R	CCAAGAGTTGCGGACAATCCAG
07g08460_F	GGGAGGAGGGCTGTATGTGAAG
07g08460_R	CTGGCTTCAGAGCCCTTCATG
07g35350_F	CGGACTGCAGTGCTATCCAACAG
07g35350_R	CATTGAATGCATAGGTCGCATGCG
07g48510_F	GGAGGTGCTGTTCTTGCAAGTG
07g48510_R	TTGGGACAAGGTCTTGGTTTGG
08g36910_F	GAGATCGATCAGTAGTGGTTAGCAG
08g36910_R	GCGTCCAGGTCGTAGAGACG
08g44390_F	GAGAAGCTCTACGCAGTCTTGG
08g44390_R	GGACAATGAAGGGAATGACCATTG
10g31850_F	GCATGGGGAAATACTTCTGCG
10g31850_R	TCGAAGCAGACAGGACAGTTG
